# Genome-wide epistatic expression quantitative trait loci discovery in four human tissues reveals the importance of local chromosomal interactions governing gene expression

**DOI:** 10.1186/s12864-015-1300-3

**Published:** 2015-02-21

**Authors:** Darren J Fitzpatrick, Colm J Ryan, Naisha Shah, Derek Greene, Cliona Molony, Denis C Shields

**Affiliations:** School of Medicine and Medical Sciences, University College Dublin, Belfield, Dublin, 4 Ireland; School of Computer Science and Informatics, University College Dublin, Belfield, Dublin, 4 Ireland; Merck Research Laboratories, Merck & Co. Inc. 33 Avenue Louis Pasteur, Boston, MA 02115 USA

## Abstract

**Background:**

Epistasis (synergistic interaction) among SNPs governing gene expression is likely to arise within transcriptional networks. However, the power to detect it is limited by the large number of combinations to be tested and the modest sample sizes of most datasets. By limiting the interaction search space firstly to *cis-trans* and then *cis-cis* SNP pairs where both SNPs had an independent effect on the expression of the most variable transcripts in the liver and brain, we greatly reduced the size of the search space.

**Results:**

Within the *cis-trans* search space we discovered three transcripts with significant epistasis. Surprisingly, all interacting SNP pairs were located nearby each other on the chromosome (within 290 kb-2.16 Mb). Despite their proximity, the interacting SNPs were outside the range of linkage disequilibrium (LD), which was absent between the pairs (r^2^ < 0.01). Accordingly, we redefined the search space to detect *cis-cis* interactions, where a *cis*-SNP was located within 10 Mb of the target transcript. The results of this show evidence for the epistatic regulation of 50 transcripts across the tissues studied. Three transcripts, namely, *HLA-G*, *PSORS1C1* and *HLA-DRB5* share common regulatory SNPs in the pre-frontal cortex and their expression is significantly correlated. This pattern of epistasis is consistent with mediation via long-range chromatin structures rather than the binding of transcription factors in *trans*. Accordingly, some of the interactions map to regions of the genome known to physically interact in lymphoblastoid cell lines while others map to known promoter and enhancer elements. SNPs involved in interactions appear to be enriched for promoter markers.

**Conclusions:**

In the context of gene expression and its regulation, our analysis indicates that the study of *cis-cis* or local epistatic interactions may have a more important role than interchromosomal interactions.

**Electronic supplementary material:**

The online version of this article (doi:10.1186/s12864-015-1300-3) contains supplementary material, which is available to authorized users.

## Background

Genome-wide studies of gene expression have successfully identified genetic variants that contribute to the variation of gene expression within populations [1-11]. The objective of genome-wide association studies (GWAS) is to map genotypic variation to phenotypic variation. Jansen and Nap [12] proposed extending the GWAS paradigm to deal with quantitative endophenotypes, e.g. RNA, protein and metabolite abundance in a cell. To date, consideration of RNA abundance has received most attention in the literature [1-11]. Those variants that affect gene expression are referred to as expression quantitative trait loci (eQTLs) of which thousands have been reported [1-11]. Most studies have focused on single nucleotide polymorphisms (SNPs).

The literature reports two classes of eQTL, *cis*-acting SNPs and *trans*-acting SNPs. *Cis*-acting SNPs lie within a gene or near the transcription start or stop site of a gene and correlate with the expression of that gene. In contrast, *trans*-acting SNPs can lie anywhere else in the genome. *Cis*-acting variants are more numerous than *trans*-acting variants but not necessarily more common due to difficulties in detecting *trans*-SNPs related to a larger search space [3-5]. Understanding of the mechanisms of action of expression polymorphisms detected in GWAS is limited. *Cis*-acting variants may affect the binding of the transcriptional machinery or the stability of the transcript [6]. The mechanics of *trans*-acting variants have proven more difficult to determine. Cheung *et al.* report that the majority of *trans*-SNPs do not map to transcription factors or signaling molecules [6]. More recently, SNPs implicated in disease associations were shown to be enriched in enhancers and microRNA binding sites [13].

As with the traditional GWAS, the focus in studies of expression polymorphisms has been the detection of single variants that function independently to affect gene expression. However, consideration of single variants alone typically explains only a small proportion of the variance of a trait. This missing heritability is attributed by some to the fact that genetic interactions (epistasis) are generally ignored in mapping studies despite claims that they are an ubiquitous feature of biological processes [14-18]. In contrast to studies in model organisms which have reported extensive epistasis [19,20], where epistasis has been studied using GWAS, the results have been few [21,22]. However, regardless of the percentage contribution to heritability, epistatic interactions are of intrinsic interest as they reveal aspects of regulatory networks that single SNPs do not identify.

The reasons for the relative absence of reported epistasis in the GWAS literature are twofold. Firstly, exhaustive searches of all pairs of SNP-SNP interactions are computationally expensive, e.g. a screen for all two-locus interactions amongst 500,000 SNPs and 30,000 transcripts would require 3.25 × 10^15^ statistical tests. More importantly, the combinatorial explosion of even simple pairwise interactions necessitates stringent correction for multiple testing which eliminates all but the most striking results. Inadequate correction for the search space and reliance on assumptions of normality in gene expression can lead to false inferences of epistasis where none exists. In this study, we sought to avoid such problems by restricting the search space in the first place, and then employing careful analyses, in particular avoiding tests that assumed normality of the quantitative RNA levels. While we do manage to reduce the search space considerably, there remains substantial lack of power to fully quantify the real extent of biologically important epistasis. Our study design is secondly limited by the availability of only four tissue types analysed on a similar platform. Nonetheless, by sampling a small fraction of such epistatic effects, our approach provides insights into the nature of epistasis governing RNA expression in mammalian tissues.

We set out to discover epistasis affecting gene expression in the human liver and three brain tissues. To reduce the size of the search space, we initially considered only variable transcripts and SNP pairs that had a *cis* and *trans* effect on the same transcript. This approach revealed a small number of statistically significant epistatic intrachromosomal effects and no interchromosomal effects. As a follow on, we redefined the search space to consider only *cis-cis* interactions. In this more focused search, we discovered a greater number of interactions affecting a greater number of transcripts, suggesting the importance of *cis-cis* epistasis in transcriptional regulation.

## Results and discussion

### Genetic interactions in four human tissues

**Figure 1:**
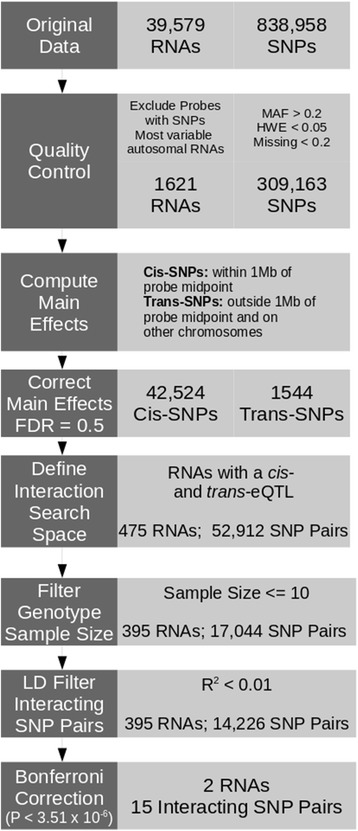
**Quality control filtering and cis-trans search space reduction of Interactions in the pre-frontal cortex.** Prior to main effect calculation, SNPs and the RNA probe sets underwent filtering for quality control. *Cis*- and *trans*-SNPs that had an independent effect on the abundance of RNA as measured on a micorarray were tested for epistatic effects on the abundance of that RNA. Only pairs of SNPs that were interchromosomal or not in linkage disequilibrium were considered. Additionally, for each of the nine genotypic combinations resulting from a pairwise interaction, only pairs that had a minimum sample size of ten individuals for each genotype were considered.

We implemented two search space strategies to detect epistatic eQTLs in the four tissues. The first strategy considered *cis-trans* interactions and the second strategy considered *cis-cis* interactions. In both cases, the criteria for testing a pairwise interaction required that the expression phenotype of a gene have both a *cis*-eQTL and a *trans*-eQTL or two *cis*-eQTLs, depending on the search space, at a liberal false discovery rate (FDR < 0.5). We excluded interacting SNP pairs in linkage disequilibrium by requiring that they have an r^2^ < 0.01. Additionally, we required that each of the nine genotypic combinations resulting from consideration of a pairwise interaction have an arbitrary minimum sample size of 10. A summary for the work flow for the *cis-trans* strategy is given in Figure 1.

### Cis-trans interactions

For the four tissues, namely, the liver, pre-frontal cortex, cerebellum and visual cortex, 1,819, 14,226, 11,562 and 4,257 tests for interaction were conducted. The differing number of tests for each tissue is partly explained by the differences in sample sizes for liver, visual cortex, cerebellum and pre-frontal cortex, i.e. 403, 403, 489 and 576 respectively. The other identifiable cause for such a difference is that the liver SNPs were genotyped on different arrays to the brain tissues. Three of the four tissues considered had epistatic eQTLs significant after Bonferroni correction ( α < 0.05), 3 in the liver, 15 in the pre-frontal cortex and 2 in the cerebellum at thresholds of p < 2.75 × 10^−5^, p < 3.51 × 10^−6^ and p < 4.32 × 10^−6^, respectively (Table 1). The interactions reported regulate an unannotated transcript on chromosome 5 and two histocompatibility genes, *HLA-G* and *HLA-DRB5* on chromosome 6. *HLA-G* is under epistatic regulation in both the liver and the pre-frontal cortex. Fitting the two main effects in the absence of an interaction term explains 7.2% - 31.10% of the phenotypic variance. When the interaction term is included, 10.6% - 33.8% of the phenotypic variance is explained with the interaction term accounting for an additional 2.8% - 5.4% of the variance. Adjusted r^2^ for the main effect models, interaction models and the likelihood ratio test statistics comparing goodness of fit are given in Additional file 1: Table S1.

**Table 1:** **Bonferroni significant cis-trans interactions in the liver, pre-frontal cortex and the cerebellum**

**Tissue**	**Cis-SNP**	**Cis position**	**Trans SNP**	**Trans position**	**Transcript**	**Interaction p-value**	**Effect size**
Liver	rs3129045	6:29652576	rs7761068	6:31333939	HLA-G	9.35 × 10^−7^	54.67
	rs2747442	6:29653186	rs7761068	6:31333939	HLA-G	2.22 × 10^−5^	47.63
	rs2747436	6:29651935	rs7761068	6:31333939	HLA-G	2.54 × 10^−5^	45.42
Pre-frontal cortex	rs9267873	6:32199352	rs2247056	6:31265490	HLA-DRB5	2.19 × 10^−8^	83.34
	rs9267873	6:32199352	rs6457374	6:31272261	HLA-DRB5	2.74 × 10^−8^	83.10
	rs507778	6:32209861	rs6457374	6:31272261	HLA-DRB5	1.57 × 10^−7^	−80.99
	rs507778	6:32209861	rs2247056	6:31265490	HLA-DRB5	2.32 × 10^−7^	−79.75
	rs2072633	6:31919578	rs3093998	6:31485174	HLA-DRB5	1.05 × 10^−7^	74.74
	rs592229	6:31930441	rs3093998	6:31485174	HLA-DRB5	2.09 × 10^−7^	72.20
	rs805262	6:31628733	rs2247056	6:31265490	HLA-DRB5	2.34 × 10^−6^	−70.73
	rs805262	6:31628733	rs6457374	6:31272261	HLA-DRB5	3.10 × 10^−6^	−70.46
	rs2858331	6:32681277	rs3093998	6:31485174	HLA-DRB5	3.24 × 10^−6^	−67.31
	rs915664	6:30794617	rs3094212	6:31085770	HLA-G	6.56 × 10^−7^	63.74
	rs13201769	6:30756066	rs2524089	6:31266522	HLA-G	7.48 x 10^−7^	−60.37
	rs13201769	6:30756066	rs2243868	6:31261276	HLA-G	8.01 × 10^−7^	−60.38
	rs10947091	6:30747216	rs2524089	6:31266522	HLA-G	9.66 × 10^−7^	−60.94
	rs10947091	6:30747216	rs2243868	6:31261276	HLA-G	1.05 × 10^−6^	−60.91
	rs3869070	6:30023868	rs3093998	6:31485714	HLA-G	1.86 × 10^−6^	66.69
Cerebellum	rs11744596	5:68519291	rs13168712	5:70679626	LOC100506658	1.52 × 10^−6^	−56.61
	rs2932777	5:68525027	rs13168712	5:70679626	LOC100506658	1.52 × 10^−6^	−56.61

Table shows the interacting *cis* and *trans* SNP identifiers, positions and two-locus p-values for the Bonferroni significant interactions in three of the four tissues studied. Groups of dependent SNPs resulting from linkage disequilibrium amongst groups of *cis*-SNPs and groups of *trans*-SNPs are demarcated by a black horizontal line.

**Figure 2:**
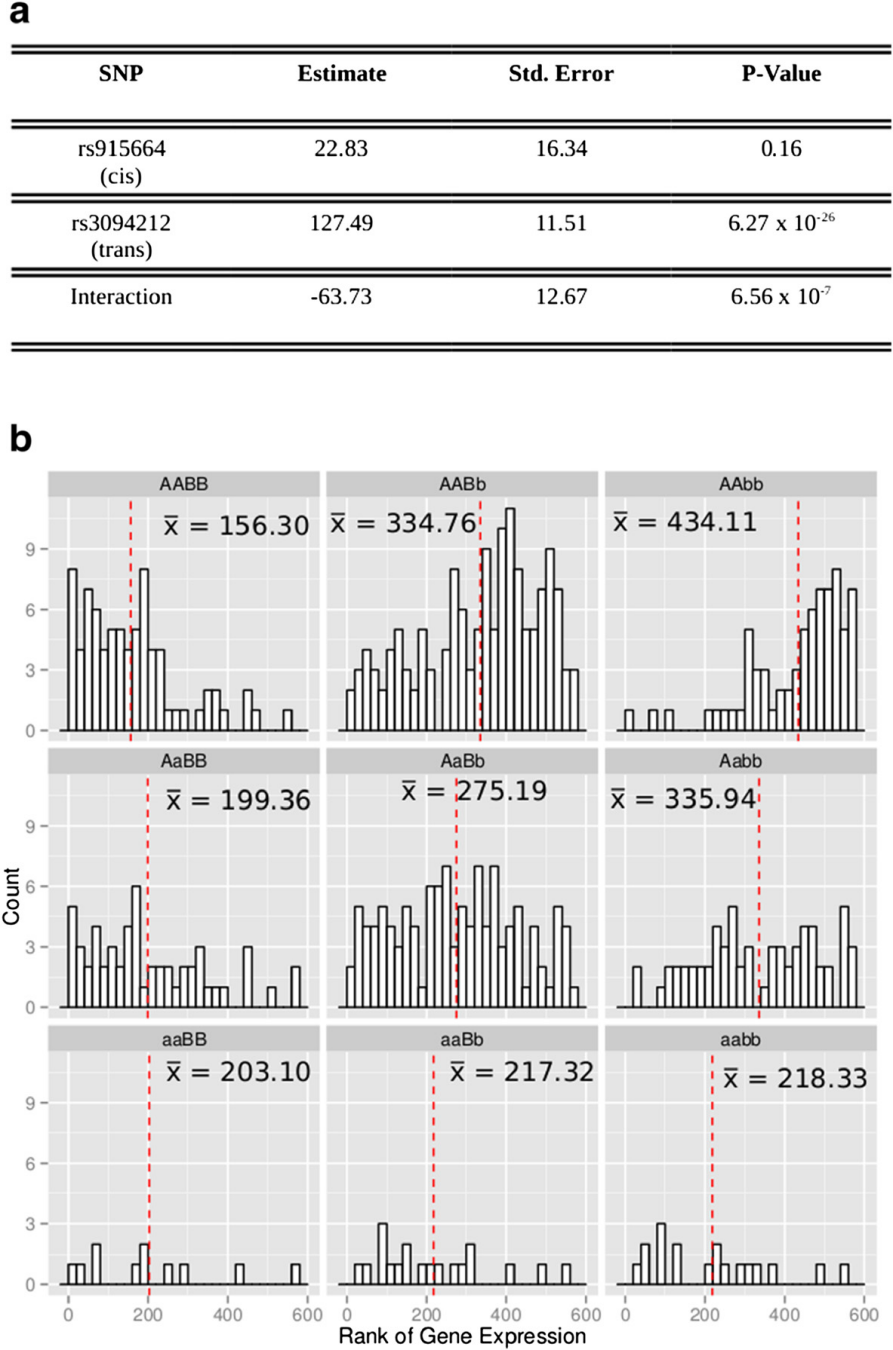
**Interaction between rs915664 (A) and rs3094212 (B) and its affect on HLA-G expression in the pre-frontal cortex. (a)** Summary of the parameters for the interaction model. **(b)** Histogram displaying the distribution of *HLA-G* gene expression (normalised to ranks, with a maximum rank of 576, representing the number of individuals in the study) stratified by pairwise genotypic combinations. The individuals with the lowest ranks have the lowest gene expression. The red line denotes the mean rank of the *HLA-G* expression for each genotype. The mean rank value is denoted xbar. Both main effects, *A* and *B* cause an increase in *HLA-G* expression as seen by the shift in means of *HLA-G* for genotypes *AABB*, *AaBB*, and *aaBB* and *AABB*, *AABb* and *AAbb*. In the absence of interaction, mean rank *HLA-G* expression for the *aabb* genotype is expected to be greater than that for the *aaBB* and *AAbb* genotypes. Consistent with the parameterisation of the model, epistasis is visible by the decrease in *HLA-G* gene expression for the *aabb* genotype.

An example of data for one of the interactions is shown in Figure 2. Figure 2b shows the distributions of the expression of *HLA-G* in the liver stratified by pairwise genotypic combinations. Both main effects, *A* and *B* cause a decrease in *HLA-G* expression as seen by the shift in means of *HLA-G* for genotypes *AABB*, *AaBB*, and *aaBB* and *ABBB*, *AABb* and *AAbb*. In the absence of interaction, mean *HLA-G* expression for the *aabb* genotype is expected to be less than that for the *aaBB* and *AAbb* genotypes. Consistent with the parameterisation of the model, epistasis is visible by the increase in *HLA-G* gene expression for the *aabb* genotype (Figure 2a). Representative interactions from the other groups reported are shown in Additional file 1: Figures S1-S9.

**Figure 3:**
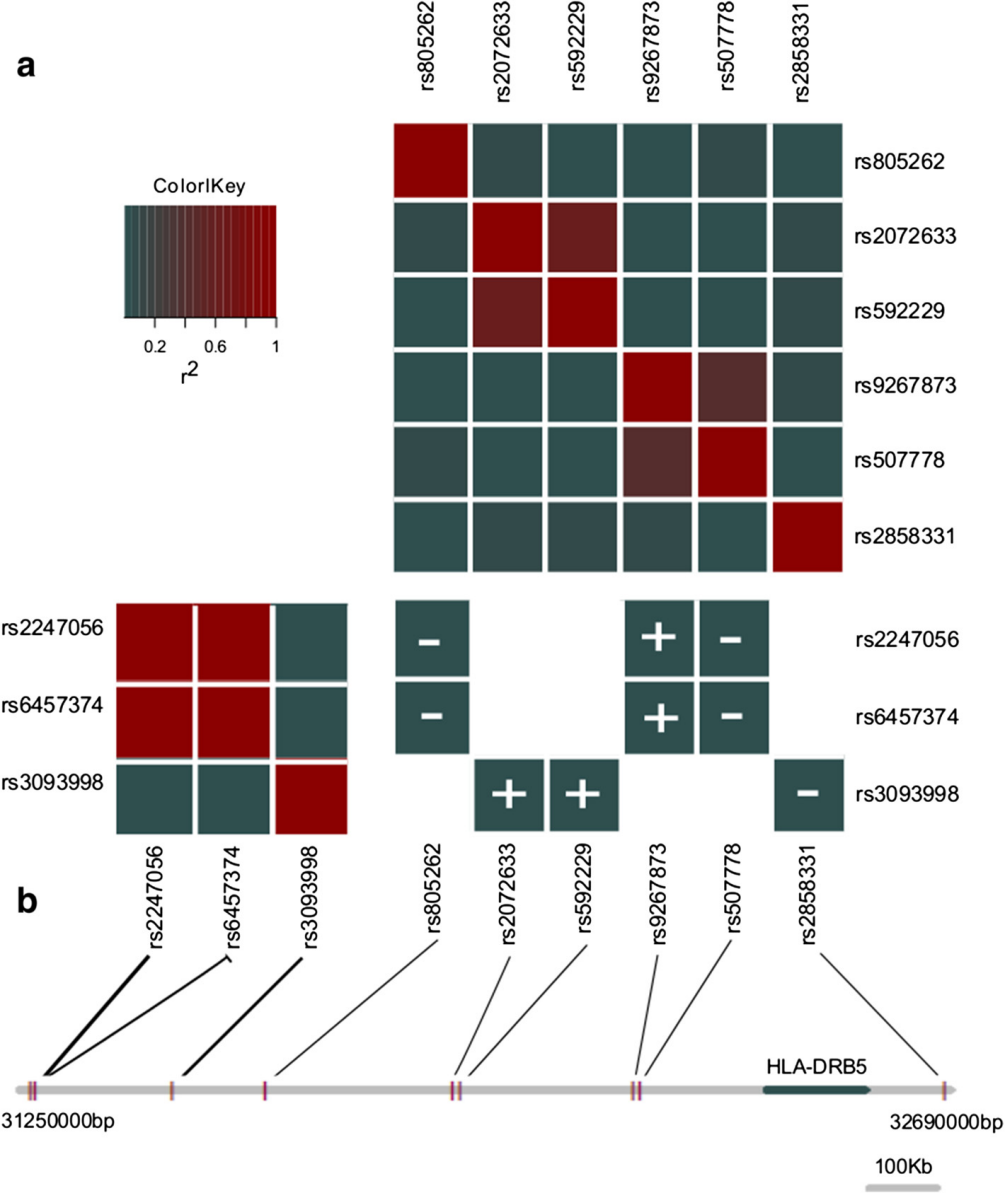
**Redundancy amongst interactions affecting HLA-DRB5 expression in the pre-frontal cortex. (a)** Linkage disequilibrium (r^2^) amongst *cis*-SNPs (top right), amongst *trans*-SNPs (bottom left) and between Bonferroni significant *cis-trans* interacting SNPs (bottom right). The interactions are denoted as positive or negative depending on the directionality of the interaction coefficient. **(b)** Schematic of the relative positions of the SNPs involved in interactions. By comparing linkage disequilibrium amongst *cis*-SNPs, amongst *trans*-SNPS and the direction of the interaction effect, the 9 interactions affecting *HLA-DRB5* collapse into 5 three groups of non-redundant interactions, (Group 1: rs9267873-rs2247056, rs9267873-rs6457374; Group 2: rs507778-rs2247056, rs507778-rs6457374; Group 3: rs805262-rs2247056, rs805262-rs6457374; Group 4: rs2072633-rs3093998, rs592229-rs3093998, Group 5: rs2858331-rs3093998).

From Table 1, it is apparent that for each significant interacting SNP pair, the *cis*- and *trans*-SNPs occupy nearby chromosomal regions. In fact, while the genome-wide screen was searching for *trans*-SNPs that lie anywhere in the genome, all of the significant findings occurred in chromosomal regions between 290 kb and 2.16 Mb from the *cis*-SNP. It would be parsimonious to assume that the pairs of interacting SNPs governing a particular transcript are essentially redundant and that once linkage disequilibrium amongst the *cis*-SNPs (and that amongst *trans*-SNPs) is accounted for, that only one interaction would remain. This is the case for an unannotated transcript on chromosome 5 expressed in the cerebellum. Once disequilibrium is accounted for at this locus, the two significant results collapse down to a single epistatic effect between a distal SNP (rs13168712) approximately 2.1 Mb apart from two proximal SNPs (rs11744596 and rs2932777) that are in complete disequilibrium. However, we found that this was only partly true. The nine significant interactions at *HLA-DRB5* reduced to five separate interactions after disequilibrium was accounted for (Figure 3). While Figure 3 illustrates that these interactions are independent of disequilibrium, we were interested to investigate if they were independent of each other. This trend was supported by a step-wise multiple regression procedure based on the Akaike Information Criterion, initially fitting all *cis* and *trans* main effect SNPs as well as the nine interaction effects detected in the screen. We noted that three of the nine interactions remained significant, indicating that there are indeed independent interaction effects at this locus (Additional file 1: Table S2). The three interactions represented three of the five groupings based on LD. It is surprising that so many independent interactions are governing a single RNA.

**Figure 4:**
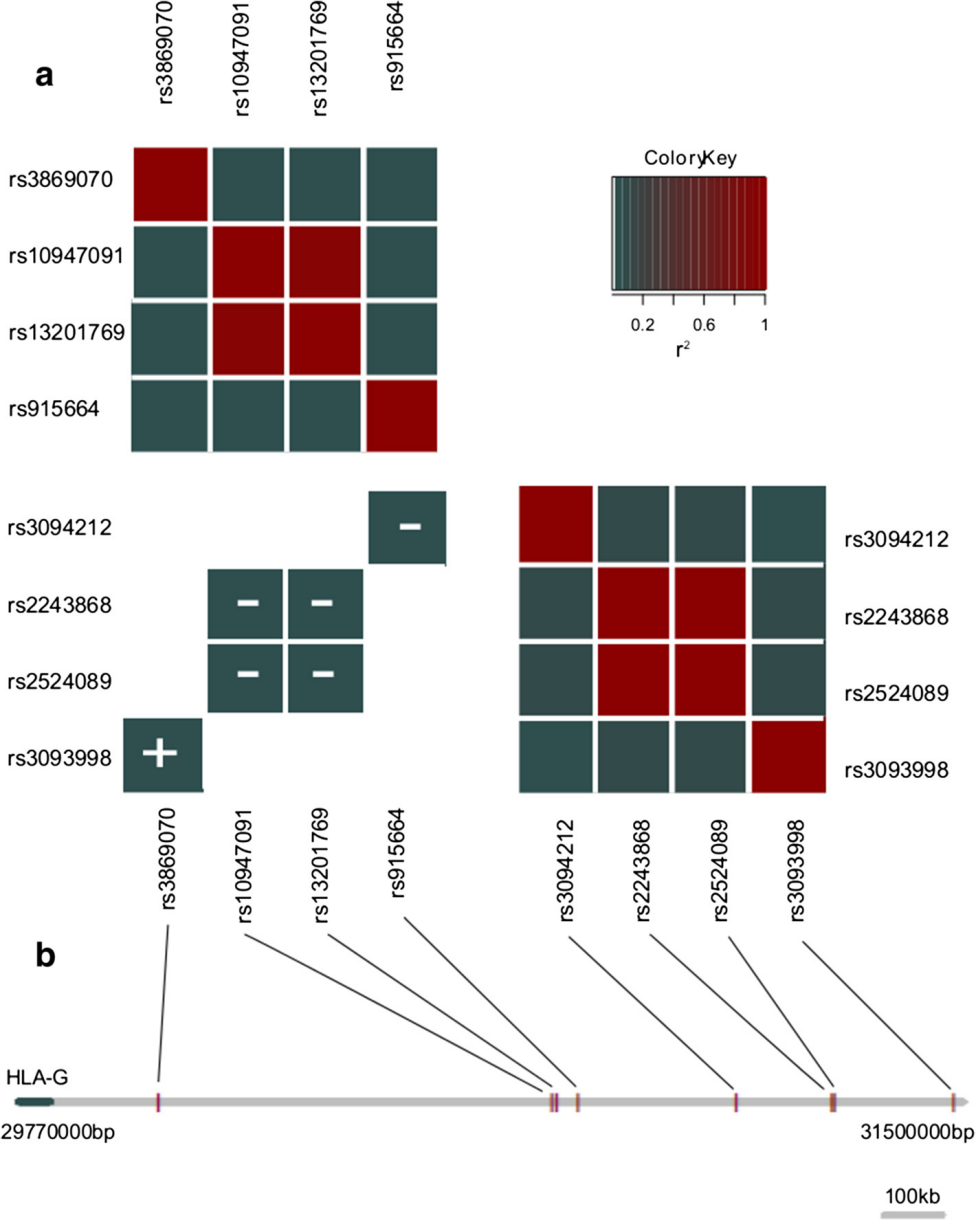
**Redundancy amongst interactions affecting HLA-G expression in the pre-frontal cortex. (a)** Linkage disequilibrium (r^2^) amongst *cis*-SNPs (top left), amongst *trans*-SNPs (bottom right) and between Bonferroni significant *cis-trans* interacting SNPs (bottom left). The interactions are denoted as positive or negative depending on the directionality of the interaction coefficient. **(b)** Schematic showing the relative proximity of SNPs involved in interactions. By comparing linkage disequilibrium amongst *cis*-SNPs, amongst *trans*-SNPS and the direction of the interaction effect, the 6 interactions affecting. *HLA-G* collapse into 3 three groups of non-redundant interactions, (Group 1: rs10947091-rs2524089, rs10947091-rs2243868, rs13201769-rs2524089, rs13201769-rs2243868; Group 2: rs915664-rs3094212; Group 3: rs3869070-3093998).

For the *HLA-G* locus, significant effects were seen in both the liver and pre-frontal cortex. The three interactions reported in the liver collapse to a single interaction. The three *cis*-SNPs are in LD (0.91 < r^2^ < 1) and interact with the same *trans*-SNP. However, the *HLA-G* locus in the pre-frontal cortex displays three distinct epistatic effects independent of LD (Figure 4) and independent of each other (Additional file 1: Table S3).

### Cis-Cis interactions

Given that the significant interactions from the *cis-trans* analysis were all intrachromosomal and that the SNPs involved were near (within 5 Mb) the transcript which they regulate, we next sought to identify such interactions by testing for *cis-cis* interactions where the *cis*-SNPs were within 10 Mb of the target transcript and of each other and at least 100 kb apart from each other and in linkage disequilibrium (r^2^ < 0.01). A total of 10,349, 135,495, 90,975 and 44,490 tests for *cis-cis* interactions were conducted in the liver, pre-frontal cortex, cerebellum and visual cortex, respectively. After Bonferroni correction ( α < 0.05), 34 interactions were significant in the liver (p < 4.83 × 10^−6^ ), 321 in the pre-frontal cortex (p < 3.69 × 10^−7^), 144 in the cerebellum (p < 5.50 × 10^−7^) and 66 in the visual cortex (p < 1.12 × 10^−6^). The interactions involve 2, 35, 27 and 16 transcripts in each of the four tissues, respectively. Details of the interactions for the four tissues are contained in Additional files 2, 3, 4, 5: Tables S4-S7.

**Figure 5:**
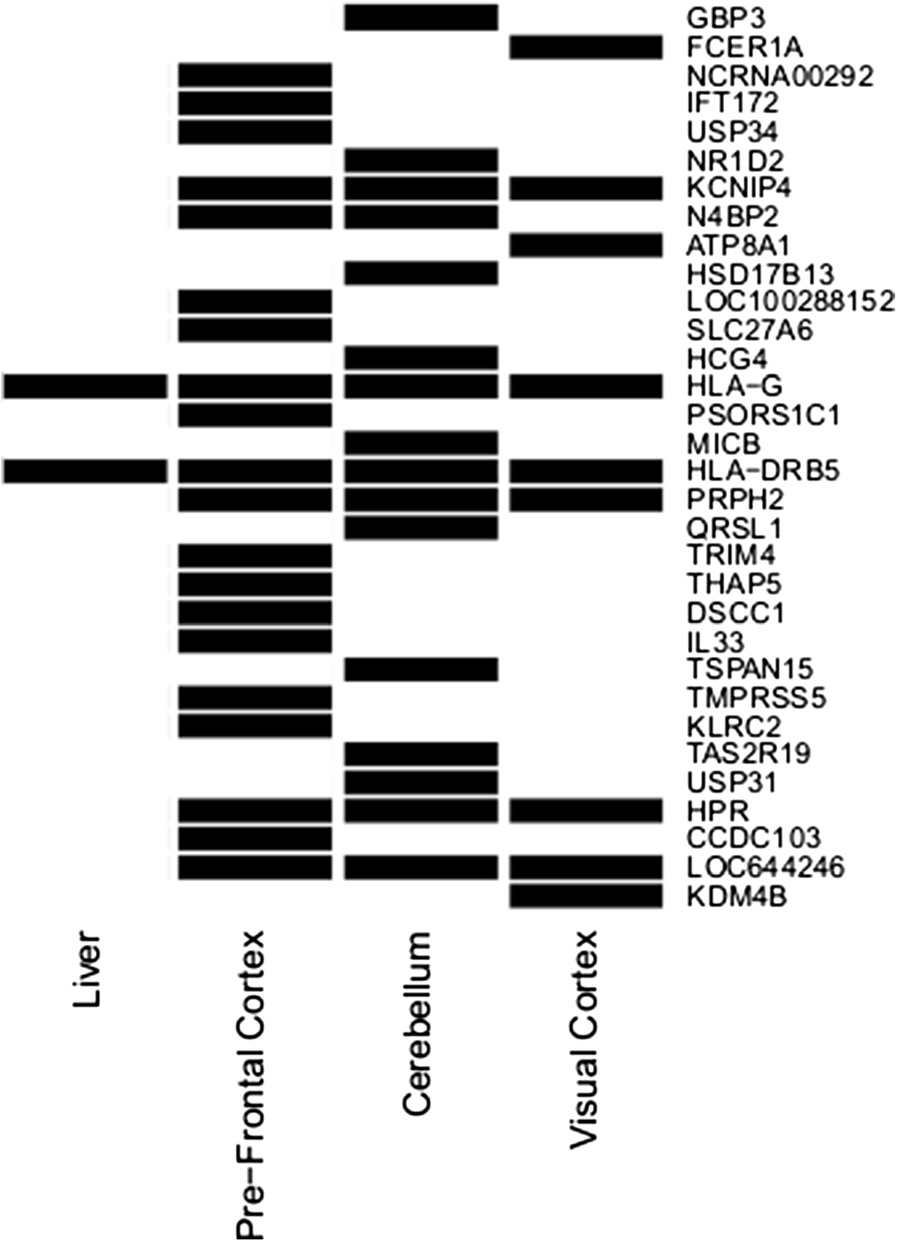
**Transcripts under epistatic regulation in the 4 tissues.** Of the 50 epistatically regulated transcripts discovered, 34 map to 32 known genes (right). The expression of 7 of these genes is under epistatic control in at least two tissues (bottom) with two genes, *HLA-DRB5* and *HLA-G* under epistatic control in all four tissues.

Of the 50 transcripts under epistatic regulation 16 are unannotated. The remaining 34 transcripts represent 32 genes. Seven of the transcripts are under epistatic regulation in at least two tissues with 2 transcripts, namely, *HLA-G* and *HLA-DRB5* under epistatic control in the liver and brain (Figure 5). Table S4 when compared with Figure 5 indicates that for some of transcripts (e.g. *NCRNA00292*, *IFT172*, *USP34*, *NR1D2*), the tissue in which the epistasis was identified was also a tissue in which the gene was more predominantly transcribed in an RNA sequencing analysis that investigated the liver, pre-fontal cortex and cerebellum [23,24]. While the observation that an RNA which is expressed more highly in a tissue may be of greater importance and more likely to come under epistatic regulatory control makes good biological sense, it must also be pointed out that the statistical power to detect epistasis is greater in more highly expressed RNAs, so this observation may also be a function of our study design. The set of epistatically controlled genes are not clearly restricted to tissue-specific genes, with a number of RNAs found to be widely expressed (Additional file 1: Table S4). For example, the *TMPRSS5* protease is the only RNA found in brain but not in kidney, heart and liver. The reported epistatic interaction affecting *HSD17B13* in the cerebellum is count-intuitive as the RNAseq study has found this RNA predominant in liver but not in the brain. A general gene ontology analysis considering all possible molecular functions did not reveal any obviously enriched set of molecular functions among the set of genes for which epistasis was observed. However, as our initial analysis highlighted the importance of the HLA region, we were interested in whether genes involved in infection or inflammation may be enriched among the remaining transcripts. We noted three gene functions that, in addition to the HLA region genes, have roles in dealing with infection, the *FECR1A* IgE receptor, the natural killer cell lectin bearing receptor *KLRC2*, and the cytokine *IL33*. Intuitively it makes good sense for genes that are involved in infection response to be more sensitive to epistasis since genetic variability in infection response is itself adaptive. It may well be that pathogen response mechanisms are enriched for epistasis. Our study is likely not to be well designed to detect such effects, since the brain is an immune privileged organ. In future it will be of interest to test formally in immune system tissues whether there is indeed a strong enrichment for epistasis in such factors.

The distance between SNPs involved in *cis-cis* interactions across the four tissues ranged from 100 kb to 1.7 Mb. Although the minimum distance allowed between interacting SNPs in the analysis was 100 kb, it is interesting that the maximum distance between two interacting SNPs was less than 2 Mb given that the maximum possible distance in the search space is 10 Mb. The empirical cumulative distributions of distances between interacting SNPs are given in Additional file 1: Figures S10-S13. Kolmogorov-Smirnov tests were used to determine whether the distribution of distances between SNPs involved in significant interactions was different from the distributions of distances between all interacting SNPs tested in each tissue. In all tissues, the two distributions were significantly different with p = 1.89 × 10^-15^ in the liver and p < 1 × 10^−16^ in the pre-frontal cortex, cerebellum and visual cortex.

In the pre-frontal cortex, there is some overlap between interactions, i.e. different transcripts share interacting SNPs. On chromosome 6 in the pre-frontal cortex, *PSORSIC1*, *HLA-G* and *HLA-DRB5* share common interactors (Additional file 1: Figure S14). The overlap of interacting SNPs could be indicative of common regulation. Consistent with this, the expression of this cluster is correlated with *HLA-G* showing correlations with *PSORS1C1* and *HLA-DRB5* (ρ = −0.21, p = 4.21 × 10^−7^; ρ = 0.18, p = 1.83 × 10^−5^, respectively) (Additional file 1: Figure S15).

### Interpreting the interactions

SNPs implicated in GWAS may not be the causal SNPs but rather linked to the causal SNP. In the case of mapping the genetic basis of disease susceptibility, if an associated SNP falls within a non-coding region, it can be difficult to decipher a mechanism of how such a SNP confers risk. With expression studies, associations link SNPs directly to the expression of a particular transcript but in itself, does not necessarily reveal anything of the transcriptional mechanism. Accordingly, we used auxiliary data in the form of the 3-dimensional structure of the genome [25] and enhancer and promoter annotations from ENCODE [26] and the Roadmap Epigenomics Project [27] in order to help interpret the functional significance of the *cis-cis* interactions reported.

### The 3-dimensional genome

The 3-dimensional structure of the genome is known to play a pivotal role in the regulation of gene expression via long-range interactions amongst enhancers, repressors and promoters [28]. These looping interactions have been characterised as part of the ENCODE project in 1% of the genome in GM12878, K562 and HeLa-S cell lines [29] and more recently in human fibroblasts [30]. Previous to this Lieberman-Aiden *et. al* reported a genome-wide map of spatial proximity using Hi-C in the GM06990 lymphoblastoid cell line [25]. We mapped the interactions reported here to pairs of spatially proximal genomic regions in the Hi-C data to identify pairs of epistatic eQTLs whose functional relationship may be mediated by long range interactions amongst regulatory elements. *Cis-cis* interactions are enriched for physical interactions in all tissues (Table 2). This enrichment of physical genomic interactions amongst epistatic interactions suggests a mechanism whereby long range intra-chromosomal eQTLs affect their target genes. However, certain caveats relating to resolution and tissue specificity apply. The mapping of SNPs to interacting Hi-C fragments is low resolution, i.e. a mapping is called if an epistatic SNP is within 5 kb of a fragment start site. Although this approach has been successful in other studies [6], the enrichment may not necessarily be due exclusively to looping interactions but to unknown features near looping interactions. Regarding tissue specificity, Dekker and colleagues, in comparing long range looping interactions in three cell lines report that although certain interactions occur across tissues, a large proportion of such interactions appear to be tissue specific [29]. For both these reasons, it is difficult to comment on the role of specific looping interactions and their effect on the expression of genes under epistatic regulation for the tissues in this study.

**Table 2:** **Epistatic interactions that map to HiC interactions**

**Tissue**	**HiC interactions**			
	**Epistatic**	**Non-epistatic**	**Odds ratio**	**P-value**
Liver	14/34	913/9772	6.79	< 0.0001
Pre-Frontal Cortex	118/321	13450/125873	4.86	< 0.0001
Cerebellum	39/105	6758/77511	6.19	< 0.0001
Visual Cortex	19/66	3422/40882	4.43	< 0.0001

SNP pairs that map to HiC interactions (spatially proximate regions of the genome) are given as the proportion of epistatic interactions that map to at least one HiC interaction and the proportion of non-epistatic SNP pairs tested that map to at least one HiC interaction. The odds ratio that a pair of epistatic SNPs also maps to a HiC physical interaction is given for each tissue. Significance is assessed using a chi-squared test for goodness of fit.

### The regulatory genome

Genomic structure can explain how seemingly distant regulatory elements are coordinated in the regulation of gene expression but regions of open chromatin including enhancer and promoter regions are a defining feature of the regulatory genome. Open chromatin is necessary for allowing regulatory DNA elements access to each other and the relevant transcriptional machinery [31]. These regulatory regions have been extensively characterised by the ENCODE project and the Roadmap Epigenomics Project in a multitude of cell types [26,27]. We mapped SNPs tested for interactions to promoter and enhancer regions in order to investigate if SNPs involved in epistatic interactions were enriched for promoter or enhancer mappings. SNPs from the liver study were mapped to promoter and enhancer annotations as measured in HepG2 cells by ENCODE and were enriched for neither promoters (OR = 1.17, P = 1) nor enhancers (OR = 0.41, P = 0.58). Likewise, SNPs from the three brain tissues were mapped to promoter and enhancer regions as measured in 7 brain tissues by the Roadmap Epigenomics Project. After correction for multiple testing, epistatic SNPs in the pre-frontal cortex were enriched for promoter mappings in 2 brain tissues and epistatic SNPs in the cerebellum were enriched for promoter mappings in 6 brain tissues (Table 3). Epistatic SNPs were not enriched for promoter mappings in the visual cortex. Similarly, enrichments were calculated for epistatic SNPs that map to enhancer regions in the same 7 brain tissues. There is no enrichment for enhancer mappings (Table 4). *A priori*, it might have been expected that very long range effects on gene expression are more likely to have enhancer marks, rather than promoter marks. It is possible that regions that engage in long range intra-chromosomal interactions have a particular distribution of histone marks that are associated with particular regulatory conformations.

**Table 3:** **SNPs that map to promoter regions in brain tissues**

**Tissue**	**Pre-frontal cortex**				**Cerebellum**				**Visual cortex**			
	**Epistatic**	**Non-epistatic**	**OR**	**P-value**	**Epistatic**	**Non-epistatic**	**OR**	**P-value**	**Epistatic**	**Non-epistatic**	**OR**	**P-value**
AC	19/258	506/19335	2.95	6.9 × 10^−6^ *	14/151	403/15883	3.93	9.75 × 10^−7^*	1/73	282/11304	0.35	0.14
HM150	18/258	463/19335	3.1	6.12 × 10^−6^*	14/151	372/15883	4.26	1.42 × 10^−7^*	3/73	265/11304	0.9	0.99
AG	13/258	424/19335	2.37	0.004	11/151	358/15883	3.41	0.00013*	3/73	251/11304	0.45	0.24
ITL	13/258	465/19335	2.15	0.012	13/151	372/15883	3.92	2.14 × 10^−6^*	3/73	273/11304	0.46	0.26
GM2	10/258	381/19335	2	0.05	9/151	326/15883	3.02	0.002	2/73	226/11304	1.02	1
MFL	15/258	421/19335	2.77	0.0002	12/151	349/15883	3.84	8.02 × 10^−6^*	3/73	250/11304	0.37	0.22
SN	15/258	485/19335	2.4	0.002	13/151	313/15883	4.69	4.68 × 10^−8^*	3/73	237/11304	0.7	0.64

SNPs that map to promoter regions as measured in 7 brain tissues by the Roadmap Epigenomics Project and mined from HaploReg are given as the proportion of SNPs that map to such regions for the set of SNPs involved in epistatic interactions and those not involved in interactions. The odds ratio (OR) that a SNP involved in an epistatic interaction will also be in an enhancer region is given. Significance is assessed using a chi-squared test for goodness of fit. Tissues with a significant enrichment of promoter mappings after Bonferroni correction for multiple testing (P < 2.4 × 10–3) are denoted with an asterisk (*).

AC – Anterior Caudate; HM150 – Hippocampus Middle; AG – Angular Gyrus; ITL – Inferior Temporal Lobe; GM2 - Germinal Matrix; MFL – Mid Frontal Lobe; SN – Substantia Nigra.

**Table 4:** **SNPs that map to enhancer regions in brain tissues**

**Tissue**	**Pre-frontal cortex**				**Cerebellum**				**Visual cortex**			
	**Epistatic**	**Non-epistatic**	**OR**	**P-value**	**Epistatic**	**Non-epistatic**	**OR**	**P-value**	**Epistatic**	**Non-epistatic**	**OR**	**P-value**
AC	23/258	1547/19335	1.12	0.67	12/151	1364/15883	1.67	0.03	3/73	928/11304	0.32	0.14
HM150	20/258	1387/19335	1.1	0.8	7/151	1200/15883	1.6	0.07	5/73	856/11304	0.9	0.99
AG	24/258	1649/19335	1.1	0.74	9/151	1439/15883	1.63	0.04	3/73	980/11304	0.45	0.24
ITL	21/258	1605/19335	0.98	1	8/151	1427/15883	0.57	0.15	3/73	959/11304	0.46	0.26
GM2	11/258	816/19335	1.01	1	5/151	702/15883	0.74	0.64	3/73	455/11304	1.02	1
MFL	14/258	1401/19335	0.73	0.32	3/151	1219/15883	0.24	0.01	2/73	802/11304	0.37	0.22
SN	26/258	1362/19335	1.48	0.08	6/151	1262/15883	0.48	0.1	4/73	857/11304	0.7	0.64

SNPs that map to enhancer regions as measured in 7 brain tissues by the Roadmap Epigenomics Project and mined from HaploReg are given as the proportion of SNPs that map to such regions for the set of SNPs involved in epistatic interactions and those not involved in interactions. The odds ratio (OR) that a SNP involved in an epistatic interaction will also be in an enhancer region is given. Significance is assessed using a chi-squared test for goodness of fit. SNPs involved in epistatic interactions are not enriched for enhancer mappings in any of the 7 brain tissues.

AC – Anterior Caudate; HM150 – Hippocampus Middle; AG – Angular Gyrus; ITL – Inferior Temporal Lobe; GM2 - Germinal Matrix; MFL – Mid Frontal Lobe; SN – Substantia Nigra.

## Conclusions

We initially conducted a genome-wide screen for *cis-trans* epistasis governing gene expression. Interestingly, all of the interactions we report using this search space are intra-chromosomal (within 290 kb - 2.16 Mb), but are not due to detectable linkage disequilibrium. Accordingly, we looked for *cis-cis* interactions and identified numerous transcripts under epistatic regulation. Such *cis-cis* genetic interactions seem important in the regulation of gene expression and appear to be a more significant contributor to large epistatic effects than inter-chromosomal and *cis-trans* effects. The patterns of multiple interactions at the *HLA* loci may be coupled with complex looping structures bringing together multiple DNA regions. Consistent with this, there is enrichment for physical interactions amongst epistatic eQTLs as well as enrichment for promoters in SNPs involved in interactions. As such, we propose that the role of distant *cis-cis* interactions in the regulation of gene expression and in disease susceptibility merits careful searching.

## Methods

### Genotype data & quality control

Individuals from the Human Liver Cohort (HLC) were genotyped on both the Affymetrix 500 K and Illumina humanHap650Y platforms. The genotyping protocol for the HLC has been described previously [5]. The Harvard Brain Tissue Resource Centre (HBTRC) samples were genotyped on both the Illumina HumanHap650Y array and a custom Perlegen 300 k array. Informed consent and ethical approval for the collection of data relating to the Human Liver Cohort was obtained from tissue resource centres at Vanderbilt University, the University of Pittsburgh and Merck Research Laboratories. All brain tissue was acquired by Merck Research Laboratories from the Harvard Brain Tissue Resource Center at McLean Hospital where informed consent was obtained from both the donors and their next of kin. The data was handled in accordance with the HBTRC guidelines. The study was approved by the McLean Hospital Institutional Review Board. For the purpose of this analysis, only SNPs on autosomes with a minor allele frequency of greater than 0.2 and a missing value quota of not more than 20% were considered. The high MAF threshold of 0.2 was used to avoid cells with small counts when investigating interactions. The highest missingness of SNPs involved in the finally reported epistatic effects was 14.64%, 4.17%, 2.66% and 2.48% in the liver, pre-frontal cortex, cerebellum and visual cortex, respectively. SNPs were filtered for violations of Hardy-Weinberg equilibrium using a Chi-Squared test (p < 0.05). After this, a total of 449,313 SNPs remained from the HLC samples and 309,976, 310,566 and 309,163 SNPs from the HBTRC cerebellum, visual cortex and pre-frontal cortex samples, respectively.

The smartpca program from the EIGENSOFT 4.2 package was used to compute principal components on the genotypic data to measure population stratification [32]. The significance (p < 0.05) of the components was determined using Tracy-Widom statistics [33]. For the liver the first principal component was statistically significant but the first three were used as covariates in all eQTL mapping analyses. For the cerebellum, visual cortex and pre-frontal cortex the first 9, 12 and 10 components were significant and used as covariates in all eQTL mapping analyses. Twenty seven individuals from the liver and 7, 6 and 7 individuals from the cerebellum, visual cortex and pre-frontal cortex were detected as outliers (smartpca default settings) and removed from all analyses. The resulting sample sizes for each of the tissues was n_liver_ = 403, n_cerebellum_ = 489, n_pre-frontal cortex_ = 576 and n_visual cortex_ = 403.

### Expression data

The liver tissue samples were collected from three tissue resource centres at Vanderbilt University, University of Pittsburgh and Merck Research Laboratories and the microarray analysis was conducted on a custom Agilent 44 k array. The expression profiling routine for the liver samples has been previously described [5]. In brief, expression is measured as the mean-log ratio relative to a sex-balanced pool of samples and adjusted for age, sex and centre of origin. The brain tissue samples were collected from the Harvard Brain Tissue Resource Centre and the expression profiling conducted on a custom Agilent array. The brain expression profiling has been described previously [34]. Similar to the liver data, the brain gene expression is measured as the mean-log ratio and has been corrected for gender, RNA integrity number, pH, post-mortem interval, batch and preservation of samples. The brain data comprised a mixture of control samples and samples with Alzheimer's and Huntington's disease.

The presence of SNPs within microarray expression probes has been shown to affect the accuracy of RNA measures [35]. We sought to remove probes containing common SNPs by mapping probes to the SNPs from the HapMap CEU population (release 128) and removing those probes containing SNPs from the analysis. In total, 6858 autosomal probes were removed.

This study considered those RNAs which were most variable in the sample populations based on the interquartile range (IQR). The interquartile range for each RNA in the four tissues was computed and the top 5% of reporters carried forward for the analyses. The IQR was chosen as a measure of variability over the variance or standard deviation so as to avoid selecting those RNAs with extreme values for few individuals. A total of 1599 reporters in the liver and 1621 reporters for each of the brain tissues were used in the analysis.

### Modeling marginal & interaction effects

Independent effects of single SNPs were estimated using rank-transform (RT) regression. RT-regression is achieved by ranking the expression values and utilising them as the dependent variable in a linear model [36]. RT-regression has been evaluated in the context of GWAS where it performs similarly to classical regression when assumptions of normality are met but has greater power and control of family-wise error rates in the presence of non-normality [37]. Genotypes *AA*, *Aa* and *aa* were encoded as 0, 1 and 2, respectively, where *A* denotes the major allele and *a*, the minor allele. Putative marginal eQTLs were designated as either *cis* or *trans* depending on their location relative to the midpoint of the expression probe coordinates. For the *cis-trans* study, *cis*-SNPs were defined as those which lie within 1 Mb upstream or downstream of the probe midpoint. Conversely, *trans*-SNPs were defined as those located outside of 1 Mb upstream or downstream of the probe midpoint or occupying a different chromosome. For the *cis-cis* study, *cis*-SNPs were defined as those SNPs which fall within 10 Mb of the probe midpoint. The putative *cis* and *trans* eQTLs were separately corrected for multiple testing using the Benjamini-Hochberg approach to control false discovery rates (FDR) [38].

Depending on the search space strategy, where an expression phenotype had both a *cis* and *trans* eQTL or two *cis*-eQTLs at an FDR of 50%, the interaction of those eQTLs was computed using a RT-regression model. Only pairs of SNPs with a minimum sample size of 10 individuals in each of the nine genotypic combinations of a pairwise interaction were considered. Fitting of both the marginal and interaction effect models was performed using R. Interactions were computed as the product of the two loci with possible values of 0, 1, 2 or 4 representing the nine genotypic combinations of a pairwise interaction. An additive model was chosen as it requires the fitting of just a single interaction term alongside the two marginal effects. The consideration of dominance would require fitting four main effects (2 additive and 2 dominant) and four interaction terms (additive*additive, dominant*dominant and 2 additive*dominant interactions) and as such require a larger sample size in order to detect such interactions. While not all epistatic effects are likely to follow an additive model, it was chosen primarily to maximise statistical power of detecting any epistatic effects. The significance of the interaction was determined using the p-value of the regression coefficient of the interaction term in the model. Interactions were corrected for multiple testing using the Bonferroni correction (α < 0.05) across all tests for that tissue. Step-wise multiple regression to determine the independence of interactions, where reported, was assessed using a step-wise multiple regression procedure based on the Akaike Information Criteria using the *step()* function in R. As an initial model, all main and interaction effect terms were fitted.

Linkage disequilibrium (LD) between interacting SNPs was measured using PLINK [39]. To ensure that interacting SNPs on the same chromosome were independent, we only tested interactions among those intra-chromosomal SNP pairs with an r^2^ < 0.01, as measured in the samples for that particular tissue. In order to determine the numbers of independent interactions, linkage disequilibrium was also measured amongst sets of significant SNP pairs that were chromosomally adjacent. Differences between the distributions of the distances between interacting SNPs and the entire set of interactions tested were tested using Kolmogorov-Smirnov statistics. The potential for the cross hybridisation of probes whose expression is under epistatic control was determined using BLAST. The probe sequence was compared to the RefSeq RNA database using a discontiguous megablast. In the case of the *cis-trans* study, no probes had a match other than itself whereas for the *cis-cis* study, 9 probes representing 5 transcripts were removed from the set of significant interactions.

### Enrichment for regulatory annotations

Promoter and enhancer annotations for *cis-cis* interacting SNPs were taken from HepG2 cells as measured by ENCODE and from 7 brain tissues (Anterior Caudate, Hippocampus Middle, Angular Gyrus, Inferior Temporal Lobe, Germinal Matrix, Mid Frontal Lobe, Substantia Nigra) as measured by the Roadmap epigenomics Project [26,27]. The annotations were gathered from HaploReg [40,41] and enrichment calculated using a Chi-squared test of independence comparing the number of SNPs involved in significant interactions that map to promoter or enhancer elements to all other SNPs tested for interactions. SNPs from the liver were tested for enrichments using the HepG2 annotations and SNPs from the three brain tissues were tested from regulatory annotations in the 7 brain tissues.

### Physical interactions from Hi-C

The genomic coordinates of interacting SNP pairs were mapped to the human genome hg18 build using the UCSC LiftOver tool [42]. Epistatic interactions were then mapped to known physically interacting regions of the genome measured in lymphoblastoid cell lines [23]. The same criteria of Cheung et al. [6] was used to map epistatic eQTLs to interacting regions, i.e. a pair of epistatic eQTLs were deemed to be in a physically interacting region of the genome when both eQTLs mapped one each +/−5 kb upstream or downstream of the alignment start sites. Enrichment for epistatic interactions were computed using a Chi-squared test of independence comparing the number of epistatic interactions that map to at least one Hi-C interaction to the number of Hi-C mappings in the remaining non-epistatic interactions tested.

### Accession codes

Microarray data for gene expression data in the liver can be downloaded from the gene expression omnibus (GEO) archive, accession no. GSE9588. Microarray data for gene expression in the pre-frontal cortex, cerebellum and visual cortex can similarly be downloaded from GEO, accession no. GSE44772, GSE44768, GSE44770, and GSE44771. The Hi-C data can also be downloaded from GEO, accession no. GSE189199.

## Additional files

Additional file 1:
**Supplementary tables and figures.**
Additional file 2: Table S4. Bonferroni significant *cis-cis* epistatic eQTLs in the liver. Table details the chromosome and position of the interacting SNPs (SNP1 and SNP2) and whether these SNPs occupy promoter and/or enhancer regions. Where a pair of interacting SNPs maps to a physically interacting HiC region, the HiC fragment is given. The statistics for the interaction models (effect size, p-value and adjusted r^2^) are given as well as the linkage disequilibrium (r^2^) between interacting SNPs. Additional file 3: Table S5. Bonferroni significant *cis-cis* epistatic eQTLs in the pre-frontal cortex. Table details the chromosome and position of the interacting SNPs (SNP1 and SNP2) and whether these SNPs occupy promoter and/or enhancer regions. Where a pair of interacting SNPs maps to a physically interacting HiC region, the HiC fragment is given. The statistics for the interaction models (effect size, p-value and adjusted r^2^) are given as well as the linkage disequilibrium (r^2^) between interacting SNPs. Additional file 4: Table S6. Bonferroni significant *cis-cis* epistatic eQTLs in the cerebellum. Table details the chromosome and position of the interacting SNPs (SNP1 and SNP2) and whether these SNPs occupy promoter and/or enhancer regions. Where a pair of interacting SNPs maps to a physically interacting HiC region, the HiC fragment is given. The statistics for the interaction models (effect size, p-value and adjusted r^2^) are given as well as the linkage disequilibrium (r^2^) between interacting SNPs. Additional file 5: Table S7. Bonferroni significant *cis-cis* epistatic eQTLs in the visual cortex. Table details the chromosome and position of the interacting SNPs (SNP1 and SNP2) and whether these SNPs occupy promoter and/or enhancer regions. Where a pair of interacting SNPs maps to a physically interacting HiC region, the HiC fragment is given. The statistics for the interaction models (effect size, p-value and adjusted r^2^) are given as well as the linkage disequilibrium (r^2^) between interacting SNPs.
