# Supplementary material for: Genome-wide epistatic expression quantitative trait loci discovery in four human tissues reveals the importance of local chromosomal interactions governing gene expression
Source: BMC Genomics. 2015 Feb 21;16(1):109. doi: 10.1186/s12864-015-1300-3 (PMC4345003; doi:10.1186/s12864-015-1300-3)

# 1 Supplementary Tables

**Table S1 - Additional details of the reported *cis-trans* interactions.**
For each interaction, the adjusted $r^2$ of the models which fit the two main effects and the models incorporating the interaction effect, the p-value of the goodness of fit as measured from a likelihood ratio test comparing both models and the measures of linkage disequilibrium ($r^2$) between interacting SNPs are given.

| Tissue | Cis-SNP | Cis Position | Trans-SNP | Trans Position | Probe I.D. | Transcript | Probe Position | Main Effects (Adj. $r^2$) | Interaction Effects (Adj$^2$) | P-Value (LRT) | LD |
|---|---|---|---|---|---|---|---|---|---|---|---|
| Liver | rs3129045 | 6:29652576 | rs7761068 | 6:31333939 | 10025907254 | HLA-G | 6:29798725-29798785 | 0.18 | 0.23 | $9.35 \times 10^{-07}$ | 0.004 |
| | rs2747442 | 6:29653186 | rs7761068 | 6: 31333939 | 10025907254 | HLA-G | 6:29798725-29798785 | 0.20 | 0.24 | $2.22 \times 10^{-05}$ | 0.005 |
| | rs2747436 | 6:29651935 | rs7761068 | 6:31333939 | 10025907254 | HLA-G | 6:29798725-29798785 | 0.18 | 0.22 | $2.54 \times 10^{-05}$ | 0.003 |
| Pre-Frontal Cortex | rs9267873 | 6:32199352 | rs2247056 | 6:31265490 | 10025904809 | HLA-DRB5 | 6:32485162-32485222 | 0.10 | 0.15 | $2.19 \times 10^{-08}$ | 0.009 |
| | rs9267873 | 6:32199352 | rs6457374 | 6:31272261 | 10025904809 | HLA-DRB5 | 6:32485162-32485222 | 0.10 | 0.15 | $2.74 \times 10^{-08}$ | 0.009 |
| | rs507778 | 6:32209861 | rs6457374 | 6:31272261 | 10025904809 | HLA-DRB5 | 6:32485162-32485222 | 0.08 | 0.13 | $1.57 \times 10^{-08}$ | 0.002 |
| | rs507778 | 6:32209861 | rs2247056 | 6:31265490 | 10025904809 | HLA-DRB5 | 6:32485162-32485222 | 0.08 | 0.12 | $2.32 \times 10^{-07}$ | 0.003 |
| | rs2072633 | 6:31919578 | rs3093998 | 6:31485174 | 10025904809 | HLA-DRB5 | 6:32485162-32485222 | 0.09 | 0.13 | $1.05 \times 10^{-07}$ | 0.007 |
| | rs592229 | 6:31930441 | rs3093998 | 6:31485174 | 10025904809 | HLA-DRB5 | 6:32485162-32485222 | 0.12 | 0.16 | $2.09 \times 10^{-07}$ | 0 |
| | rs805262 | 6:31628733 | rs2247056 | 6:31265490 | 10025904809 | HLA-DRB5 | 6:32485162-32485222 | 0.07 | 0.12 | $2.34 \times 10^{-06}$ | 0 |
| | rs805262 | 6:31628733 | rs6457374 | 6:31272261 | 10025904809 | HLA-DRB5 | 6:32485162-32485222 | 0.07 | 0.10 | $3.10 \times 10^{-06}$ | 0 |
| | rs2858331 | 6:32681277 | rs3093998 | 6:31485174 | 10025904809 | HLA-DRB5 | 6:32485162-32485222 | 0.07 | 0.11 | $3.24 \times 10^{-06}$ | 0.008 |
| | rs915664 | 6:30794617 | rs3094212 | 6:31085770 | 10025907254 | HLA-G | 6:29798725-29798785 | 0.24 | 0.27 | $6.56 \times 10^{-07}$ | 0.004 |
| Cerebellum | rs13201769 | 6:30756066 | rs2524089 | 6:31266522 | 10025907254 | HLA-G | 6:29798725-29798785 | 0.31 | 0.34 | $7.48 \times 10^{-07}$ | 0.001 |
| | rs13201769 | 6:30756066 | rs2243868 | 6:31261276 | 10025907254 | HLA-G | 6:29798725-29798785 | 0.31 | 0.34 | $8.01 \times 10^{-07}$ | 0.001 |
| | rs10947091 | 6:30747216 | rs2524089 | 6:31266522 | 10025907254 | HLA-G | 6:29798725-29798785 | 0.30 | 0.33 | $9.66 \times 10^{-07}$ | 0.001 |
| | rs10947091 | 6:30747216 | rs2243868 | 6:31261276 | 10025907254 | HLA-G | 6:29798725-29798785 | 0.30 | 0.33 | $1.05 \times 10^{-06}$ | 0.001 |
| | rs3869070 | 6:30023868 | rs3093998 | 6:31485174 | 10025907254 | HLA-G | 6:29798725-29798785 | 0.13 | 0.17 | $1.86 \times 10^{-06}$ | 0.008 |
| | rs11744596 | 5:68519291 | rs13168712 | 5:70679626 | 10023810270 | LOC100506658 | 5:68849630-68849690 | 0.21 | 0.25 | $1.52 \times 10^{-06}$ | 0.005 |
| | rs2932777 | 5:68525027 | rs13168712 | 5:70679626 | 10023810270 | LOC100506658 | 5:68849630-68849690 | 0.21 | 0.25 | $1.52 \times 10^{-06}$ | 0.005 |

**Table S2 - Multiple Regression Model of Main Effect and Interaction Terms Showing Independent Effects on HLA-DRB5 Expression in the Pre-Frontal Cortex.**

| Model Terms | Effect Size | P-Value |
|---|---|---|
| rs2072633 | -55.675 | 0.001997 |
| rs2858331 | 26.475 | 0.053860 |
| rs592229 | 58.684 | $7.42 \times 10^{-05}$ |
| rs9267873 | 4.250 | 0.735505 |
| rs2247056 | -26.552 | 0.126733 |
| rs3093998 | 38.964 | 0.057787 |
| rs9267873:rs2247056 | 56.654 | 0.000139 |
| rs2072633:rs3093998 | 39.399 | 0.007252 |
| rs2858331:rs3093998 | -40.709 | 0.005094 |

**Table S3 - Multiple Regression Model of Main Effect and Interaction Terms Showing Independent Effects on HLA-G Expression in the Pre-Frontal Cortex.**

| Model Terms | Effect Size | P-Value |
|---|---|---|
| rs10947091 | 87.249 | 0.028331 |
| rs13201769 | -109.688 | 0.004251 |
| rs3869070 | -11.171 | 0.336558 |
| rs915664 | 20.560 | 0.166855 |
| rs2524089 | 88.493 | 1.22e-11 |
| rs3093998 | -6.221 | 0.660640 |
| rs3094212 | 74.164 | 5.85e-10 |
| rs915664:rs3094212 | -39.636 | 0.000911 |
| rs10947091:rs2524089 | -29.304 | 0.018983 |
| rs3869070:rs3093998 | 33.367 | 0.005853 |

**Table S4 - RNA-Seq derived expression data from Brawand *et al* (2011). The RNA-Seq data is givien as the $\log_2$ tranformed reads per kilobase of transcript per million mapped reads (RPKM). The tissues of relevance for this study are the liver, pre-frontal cortex and cerebellum. RNA-Seq data for 26 of the 32 transcripts reported to be under epistatic control in this study. Values in bold indicate the tissue in which a gene is reported to be regulated by epistasis. Where expression data was not available, this is indicated by a dash (-)**

| Gene | Liver | Pre-Frontal Cortex | Cerebellum | Frontal Lobe | Temporal Lobe | Heart | Kidney | Testis |
|---|---|---|---|---|---|---|---|---|
| MICB | - | - | **4** | 0.7 | 0.6 | 1 | 1 | 3 |
| KLRC2 | - | **0.9** | - | - | 0.9 | - | - | - |
| HSD17B13 | 132 | - | **-** | - | - | - | - | - |
| THAP5 | 3 | **5** | 3 | 2 | - | 1 | 2 | 6 |
| IL33 | 2 | 3 | **0.9** | 1 | - | 4 | - | 3 |
| KCNIP4 | - | **27** | **10** | 13 | 3 | - | 3 | - |
| CCDC103 | - | **-** | - | - | - | - | - | 44 |
| N4BP2 | - | **-** | **-** | - | - | - | - | 0.9 |
| QRSL1 | 4 | 3 | **1** | 2 | 1 | 3 | 3 | 4 |
| TRIM4 | 3 | **3** | 6 | 2 | 2 | 3 | 7 | 8 |
| NR1D2 | 5 | 21 | **39** | 16 | 12 | 3 | 7 | 4 |
| HLA-DRB5 | **6** | **2** | **1** | 6 | 1 | 6 | 13 | 70 |
| USP34 | 7 | **22** | 11 | 8 | 4 | 6 | 6 | 17 |
| USP31 | 5 | 12 | **13** | 12 | 10 | 9 | 9 | 24 |
| FCER1A | 2 | - | - | - | - | - | - | 0.8 |
| NCR-00292 | 0.8 | **2** | 0.8 | 2 | 2 | - | 0.9 | 2 |
| TSPAN15 | 6 | 11 | **48** | 78 | 79 | 11 | 19 | 40 |
| TMPRSS5 | - | **3** | 2 | 4 | 7 | - | - | 2 |
| PRPH2 | - | **7** | **0.7** | 2 | 4 | - | 0.6 | 2 |
| HPR | - | **1** | **4** | 1 | 6 | 47 | 14 | 2 |
| HLA-G | **1** | **0.9** | **-** | 0.7 | 1 | - | 5 | 7 |
| IFT172 | 3 | **25** | 23 | 14 | 19 | 11 | 17 | 76 |
| PSORS1C1 | - | **-** | - | 2 | - | 0.6 | - | 82 |
| KDM4B | 16 | 6 | 18 | 10 | 52 | 9 | 6 | 35 |
| SLC27A6 | - | **-** | - | 1 | 0.6 | 4 | - | 2 |
| HCG4 | - | 0.9 | **1** | 0.8 | - | - | 1 | 0.9 |
| GBP3 | 2 | 2 | **1** | 1 | 0.8 | 4 | 7 | 2 |
| ATP8A1 | - | 25 | 8 | 35 | 7 | 2 | 0.7 | 0.6 |
| DSCC1 | 0.6 | **0.7** | 2 | 1 | 2 | - | - | 3 |

## 2 Supplementary Figures

**Figure S1 - Interaction between rs507778 (A) and rs6457374 (B) and its affect on HLA-DRB5 expression in the pre-frontal cortex.**

(a) Summary of the parameters for the interaction model. (b) Distribution of HLA-DRB5 expression stratified by pairwise genotypic combinations. The red line denotes the mean of the ranked HLA-DRB5 expression for each genotype. The mean value is denoted $\bar{x}$. The mean rank expression for the double homozygote (aabb) is less than would be expected if no interaction were present and both SNPs had independent effects on the expression of HLA-DRB5.

(a)

| SNP | Estimate | Std. Error | P-Value |
|---|---|---|---|
| rs507778 (cis) | 6.49 | 12.85 | 0.61 |
| rs6457374 (trans) | 124.89 | 16.09 | $4 \times 10^{-14}$ |
| Interaction | -80.99 | 15.25 | $1.57 \times 10^{-7}$ |

(b)

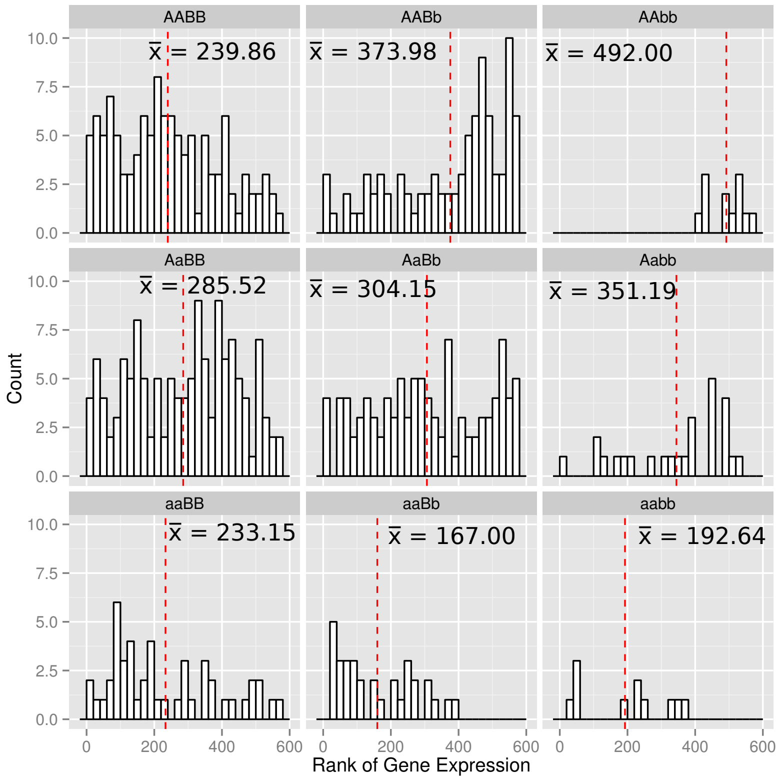

**Figure S2 - Interaction between rs2072633 (A) and rs3093998 (B) and its affect on HLA-DRB5 expression in the pre-frontal cortex.**

(a) Summary of the parameters for the interaction model. (b) Distribution of HLA-DRB5 expression stratified by pairwise genotypic combinations. The red line denotes the mean of the ranked HLA-DRB5 expression for each genotype. The mean value is denoted $\bar{x}$. The mean rank expression for the double homozygote (aabb) is greater than would be expected if no interaction were present and both SNPs had independent effects on the expression of HLA-DRB5.

(a)

| SNP | Estimate | Std. Error | P-Value |
|---|---|---|---|
| rs2072633 (cis) | -12.53 | 13.90 | 0.37 |
| rs3093998 (trans) | -5.96 | 14.94 | 0.69 |
| Interaction | 74.74 | 13.87 | $1.05 \times 10^{-7}$ |

(b)

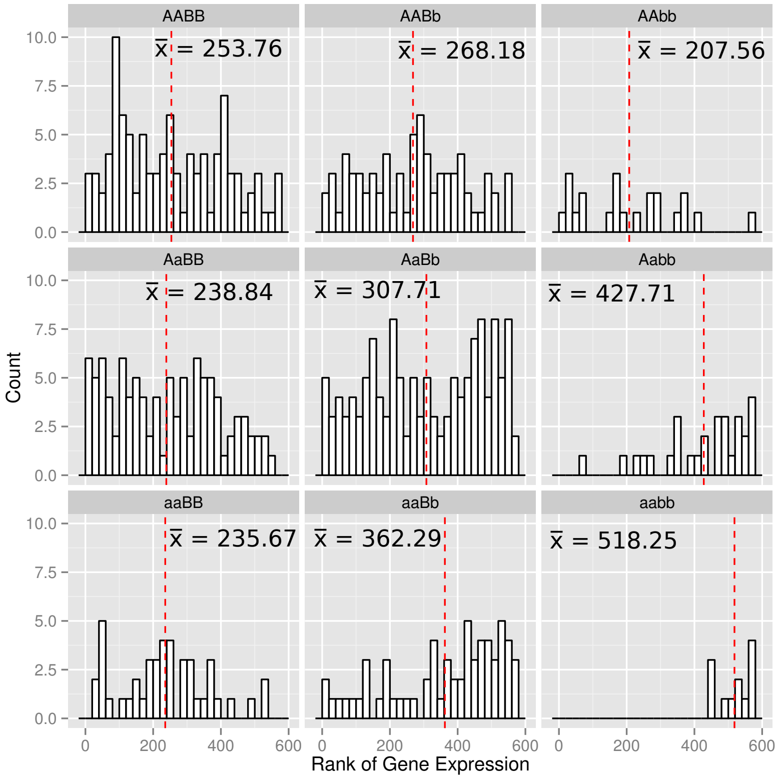

**Figure S3 - Interaction between rs805262 (A) and rs2247056 (B) and its affect on HLA-DRB5 expression in the pre-frontal cortex.**

(a) Summary of the parameters for the interaction model. (b) Distribution of HLA-DRB5 expression stratified by pairwise genotypic combinations. The red line denotes the mean of the ranked HLA-DRB5 expression for each genotype. The mean value is denoted $\bar{x}$. The mean rank expression for the double homozygote (aabb) is less than would be expected if no interaction were present and both SNPs had independent effects on the expression of HLA-DRB5.

(a)

| SNP | Estimate | Std. Error | P-Value |
|---|---|---|---|
| rs805262 (cis) | 11.41 | 12.42 | 0.36 |
| rs2247056 (trans) | 122.28 | 17.07 | $2.55 \times 10^{-12}$ |
| Interaction | 72.20 | 14.83 | $2.34 \times 10^{-6}$ |

(b)

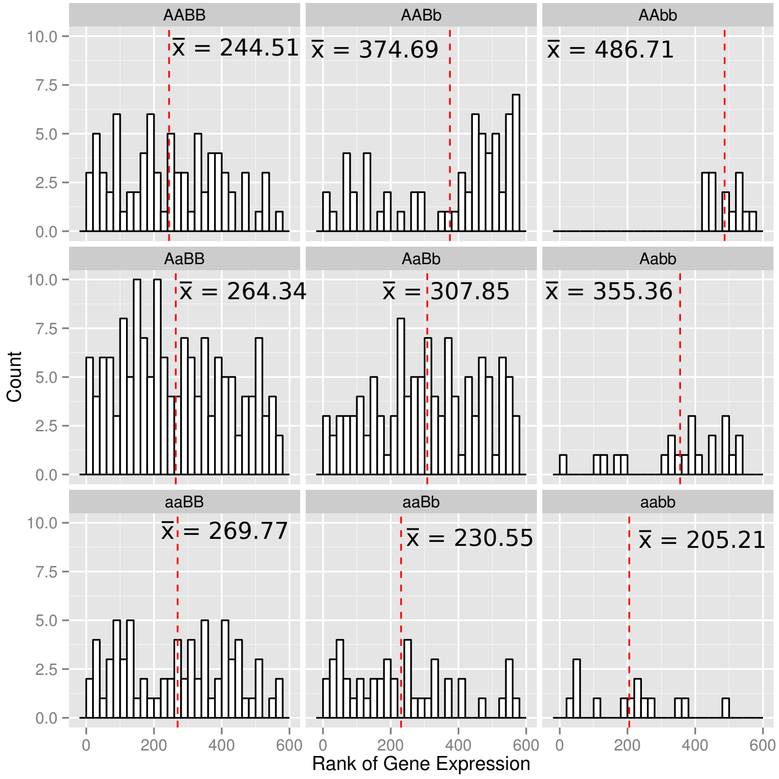

**Figure S4 - Interaction between rs2858331 (A) and rs3093998 (B) and its affect on HLA-DRB5 expression in the pre-frontal cortex.**

(a) Summary of the parameters for the interaction model. (b) Distribution of HLA-DRB5 expression stratified by pairwise genotypic combinations. The red line denotes the mean of the ranked HLA-DRB5 expression for each genotype. The mean value is denoted $\bar{x}$. The mean rank expression for the double homozygote (aabb) is less than would be expected if no interaction were present and both SNPs had independent effects on the expression of HLA-DRB5.

(a)

| SNP | Estimate | Std. Error | P-Value |
|---|---|---|---|
| rs2858331 (cis) | 24.51 | 13.96 | 0.08 |
| rs3093998 (trans) | 108.09 | 14.96 | $1.64 \times 10^{-12}$ |
| Interaction | -67.31 | 14.31 | $3.24 \times 10^{-6}$ |

(b)

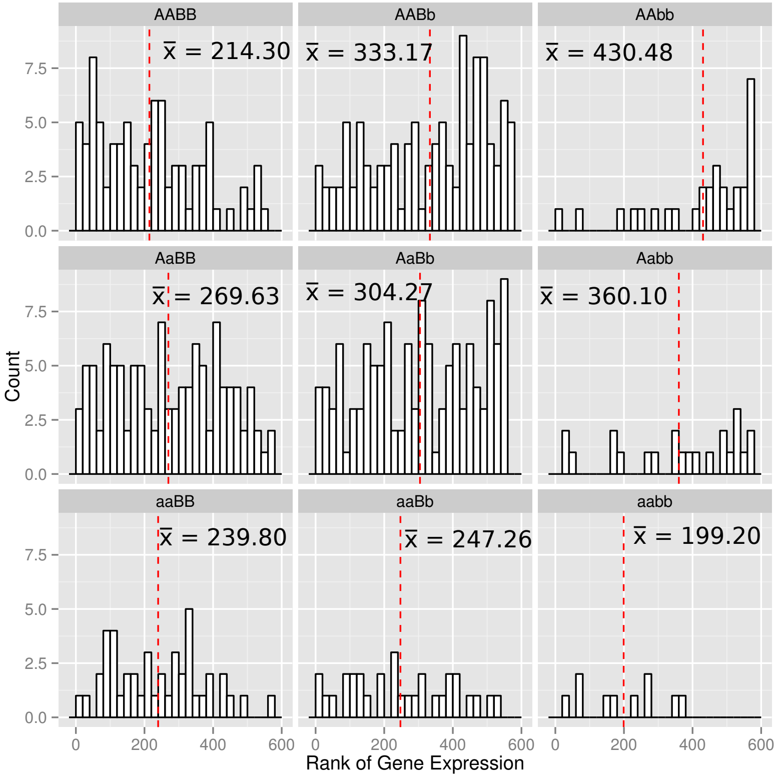

**Figure S5 - Interaction between rs915664 (A) and rs3094212 (B) and its affect on HLA-G expression in the pre-frontal cortex.**

(a) Summary of the parameters for the interaction model. (b) Distribution of HLA-G expression stratified by pairwise genotypic combinations. The red line denotes the mean of the ranked HLA-G expression for each genotype. The mean value is denoted $\bar{x}$. The mean rank expression for the double homozygote (aabb) is less than would be expected if no interaction were present and both SNPs had independent effects on the expression of HLA-G.

(a)

| SNP | Estimate | Std. Error | P-Value |
|---|---|---|---|
| rs915664 (cis) | 22.83 | 16.34 | 0.16 |
| rs3094212 (trans) | 127.49 | 11.51 | $6.27 \times 10^{-26}$ |
| Interaction | -63.73 | 12.67 | $6.56 \times 10^{-7}$ |

(b)

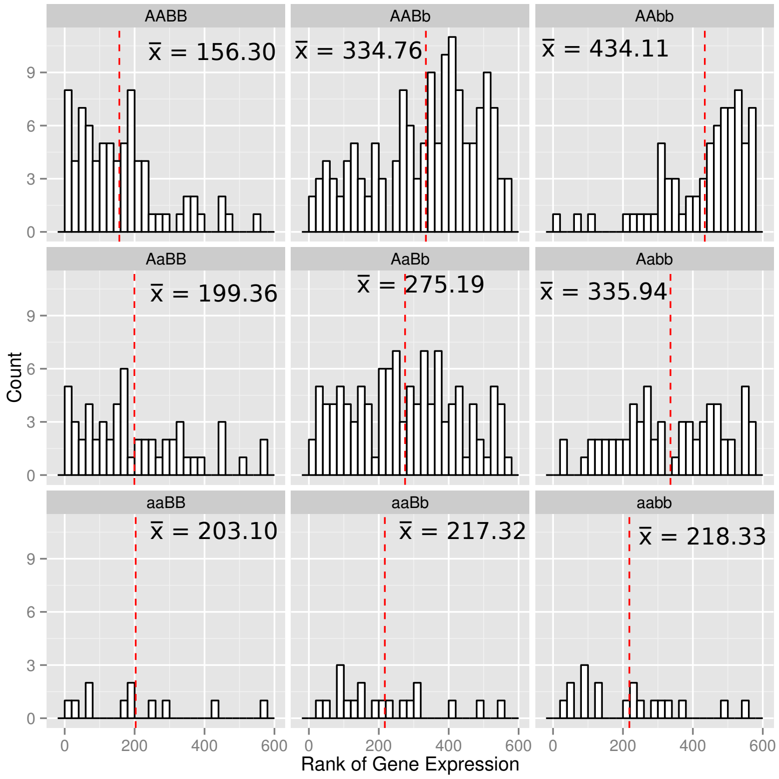

**Figure S6 - Interaction between rs13201769 (A) and rs2524089 (B) and its affect on HLA-G expression in the pre-frontal cortex.**

(a) Summary of the parameters for the interaction model. (b) Distribution of HLA-G expression stratified by pairwise genotypic combinations. The red line denotes the mean of the ranked HLA-G expression for each genotype. The mean value is denoted $\bar{x}$. The mean rank expression for the double homozygote (aabb) is less than would be expected if no interaction were present and both SNPs had independent effects on the expression of HLA-G.

(a)

| SNP | Estimate | Std. Error | P-Value |
|---|---|---|---|
| rs13201769 (cis) | -14.42 | 13.17 | 0.27 |
| rs2524089 (trans) | 139.07 | 11.59 | $1.17 \times 10^{-29}$ |
| Interaction | -60.37 | 12.06 | $7.48 \times 10^{-7}$ |

(b)

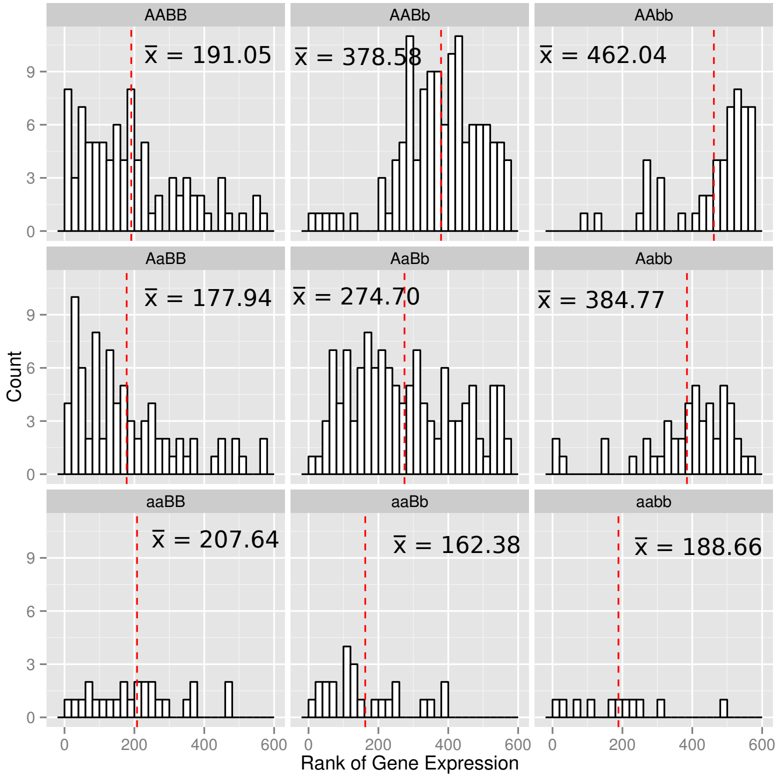

**Figure S7 - Interaction between rs3129045 (A) and rs7761068 (B) and its affect on HLA-G expression in the liver.**

(a) Summary of the parameters for the interaction model. (b) Distribution of *HLA-G* expression stratified by pairwise genotypic combinations. The red line denotes the mean of the ranked HLA-G expression for each genotype. The mean value is denoted $\bar{x}$. Both main effects, A and B cause a decrease in *HLA-G* expression as seen by the shift in means of *HLA-G* for genotypes AABB, AaBB, and aaBB and ABBB, AABb and AAbb. In the absence of interaction, mean *HLA-G* expression for the aabb genotype is expected to be less than that for the aaBB and AAbb genotypes. Consistent with the parameterisation of the model, epistasis is visible by the increase in *HLA-G* gene expression for the aabb genotype.

(a)

| SNP | Estimate | Std. Error | P-Value |
|---|---|---|---|
| rs3129045 (cis) | -97.48 | 13.63 | $5.36 \times 10^{-12}$ |
| rs7761068 (trans) | -98.47 | 11.30 | $1.32 \times 10^{-16}$ |
| Interaction | 54.67 | 10.94 | $9.35 \times 10^{-7}$ |

(b)

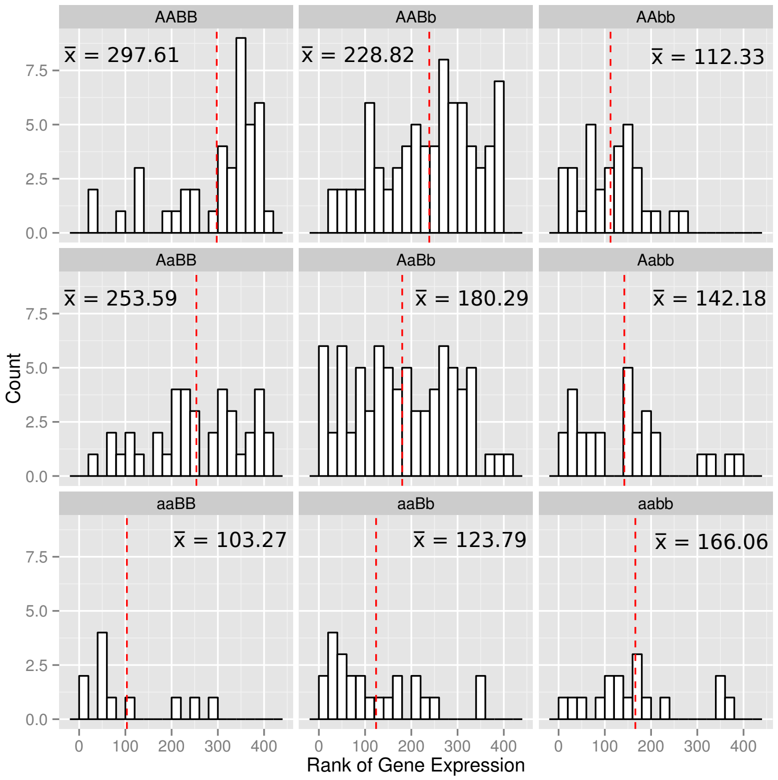

**Figure S8 - Interaction between rs3869070 (A) and rs3093998 (B) and its affect on HLA-G expression in the pre-frontal cortex.**

(a) Summary of the parameters for the interaction model. (b) Distribution of HLA-G expression stratified by pairwise genotypic combinations. The red line denotes the mean of the ranked HLA-G expression for each genotype. The mean value is denoted $\bar{x}$. The mean rank expression for the double homozygote (aabb) is greater than would be expected if no interaction were present and both SNPs had independent effects on the expression of HLA-G.

(a)

| SNP | Estimate | Std. Error | P-Value |
|---|---|---|---|
| rs3869070 (cis) | -26.46 | 13.54 | 0.05 |
| rs3093998 (trans) | -7.20 | 15.86 | 0.65 |
| Interaction | 66.69 | 13.84 | $6.56 \times 10^{-7}$ |

(b)

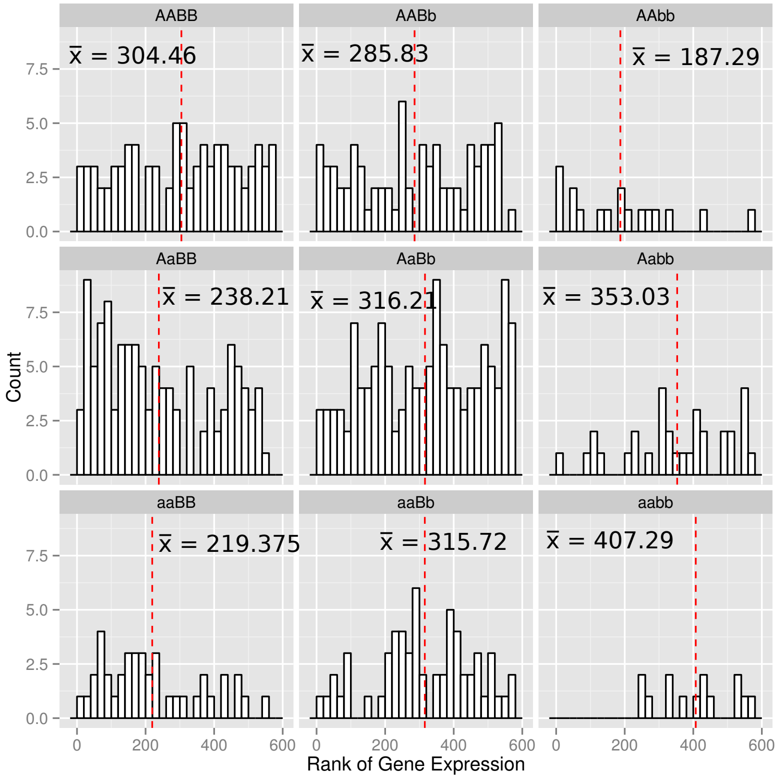

**Figure S9 - Interaction between rs11744596 (A) and rs13168712 (B) and its affect on LOC100506658 expression in the cerebellum.**

(a) Summary of the parameters for the interaction model. (b) Distribution of LOC100506658 expression stratified by pairwise genotypic combinations. The red line denotes the mean of the ranked LOC100506658 expression for each genotype. The mean value is denoted $\bar{x}$. The mean rank expression for the double homozygote (aabb) is less than would be expected if no interaction were present and both SNPs had independent effects on the expression of LOC100506658.

(a)

| SNP | Estimate | Std. Error | P-Value |
|---|---|---|---|
| rs11744596 (cis) | 74.81 | 11.52 | $2.11 \times 10^{-10}$ |
| rs13168712 (trans) | 130.94 | 12.81 | $2.67 \times 10^{-22}$ |
| Interaction | -56.61 | 11.62 | $1.52 \times 10^{-6}$ |

(b)

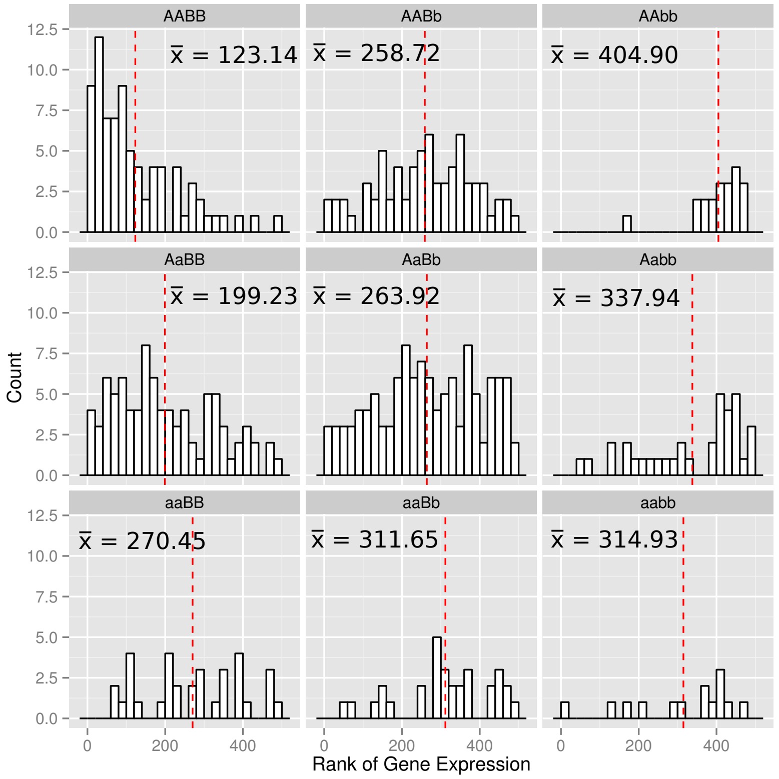

**Figure S10 - Empirical cumulative distribution of the distances between SNPs in significant interactions (red) and all SNPs tested for interaction (black) in the liver.**

The distributions of distances between SNPs involved in interaction versus the entire set of interactions tested are different from each other ($p = 1.89 \times 10^{-15}$).

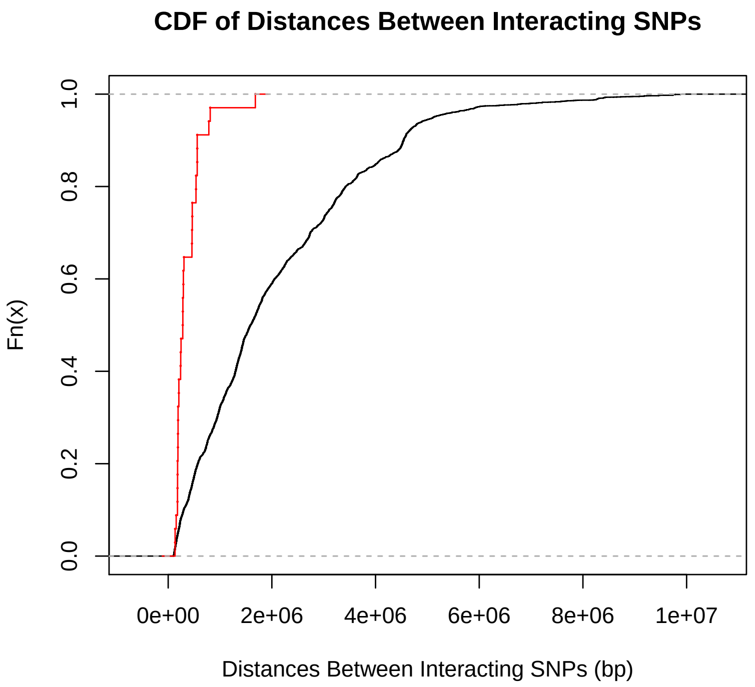

**Figure S11 - Empirical cumulative distribution of the distances between SNPs in significant interactions (red) and all SNPs tested for interaction (black) in the pre-frontal cortex.**
The distributions of distances between SNPs involved in interaction versus the entire set of interactions tested are different from each other ($p < 1 \times 10^{-16}$)

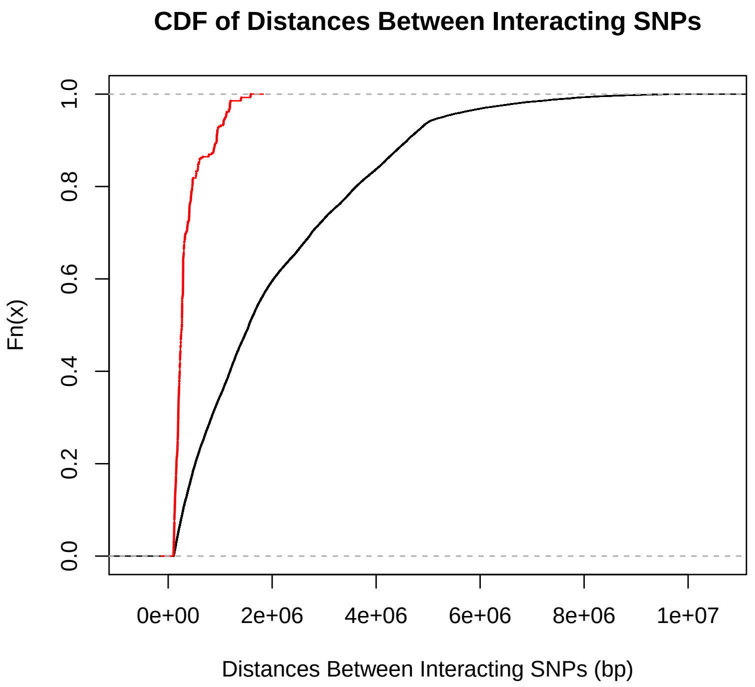

**Figure S12 - Empirical cumulative distribution of the distances between SNPs in significant interactions (red) and all SNPs tested for interaction (black) in the cerebellum.**
The distributions of distances between SNPs involved in interaction versus the entire set of interactions tested are different from each other ($p < 1 \times 10^{-16}$)

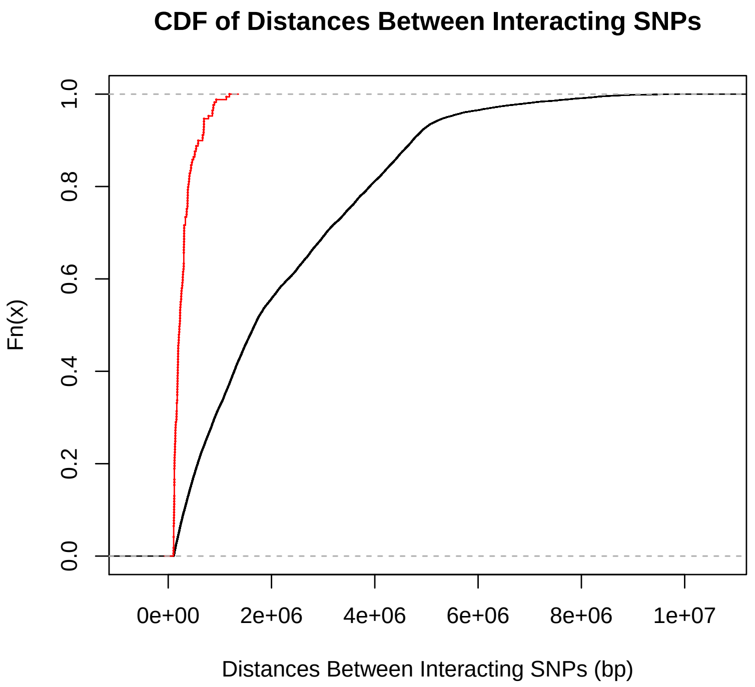

**Figure S13 - Empirical cumulative distribution of the distances between SNPs in significant interactions (red) and all SNPs tested for interaction (black) in the visual cortex.**
The distributions of distances between SNPs involved in interaction versus the entire set of interactions tested are different from each other ($p < 1 \times 10^{-16}$)

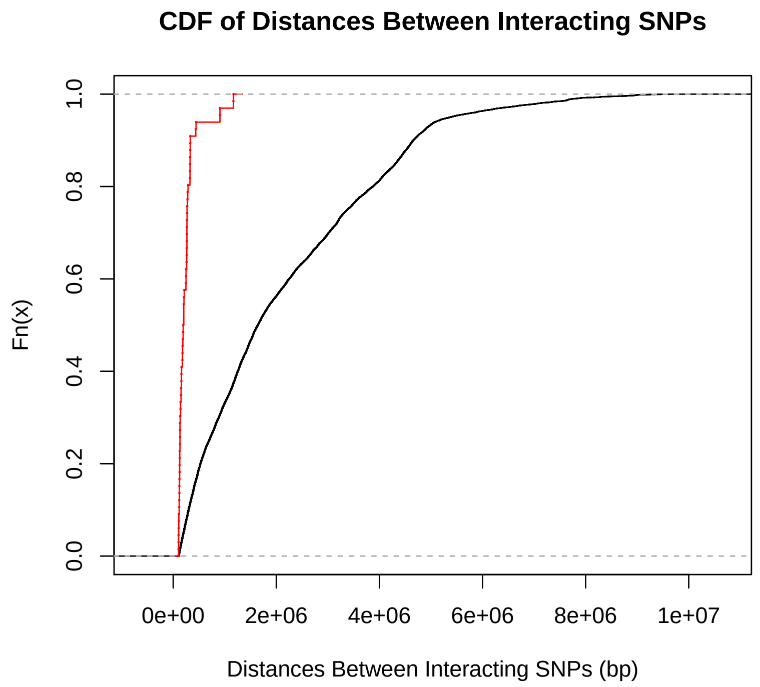

**Figure S14 - HLA-G, PSORS1C1 and HLA-DRB5 share common interactors in the pre-frontal cortex.** SNPs involved in interactions affecting the expression of any of *HLA-G*, *PSORS1C1* or *HLA-DRB5* are denoted by a black rectangle. Where multiple rectangles exist for a single SNP, that SNP is a common interactor, i.e., rs2240064 is involved in interactions affecting the expression of *HLA-G* and *PSORS1C1*. SNPs are ordered from left to right in increasing order of their position on chromosome 6. The common interactors of the three transcripts span $\approx$ 1.6Mb from rs2240064 (chr6:31114573) to rs2858331 (chr6:32681277). The schematic (bottom) shows the relative posiiton of the three genes and the common interactors on chromosome 6.

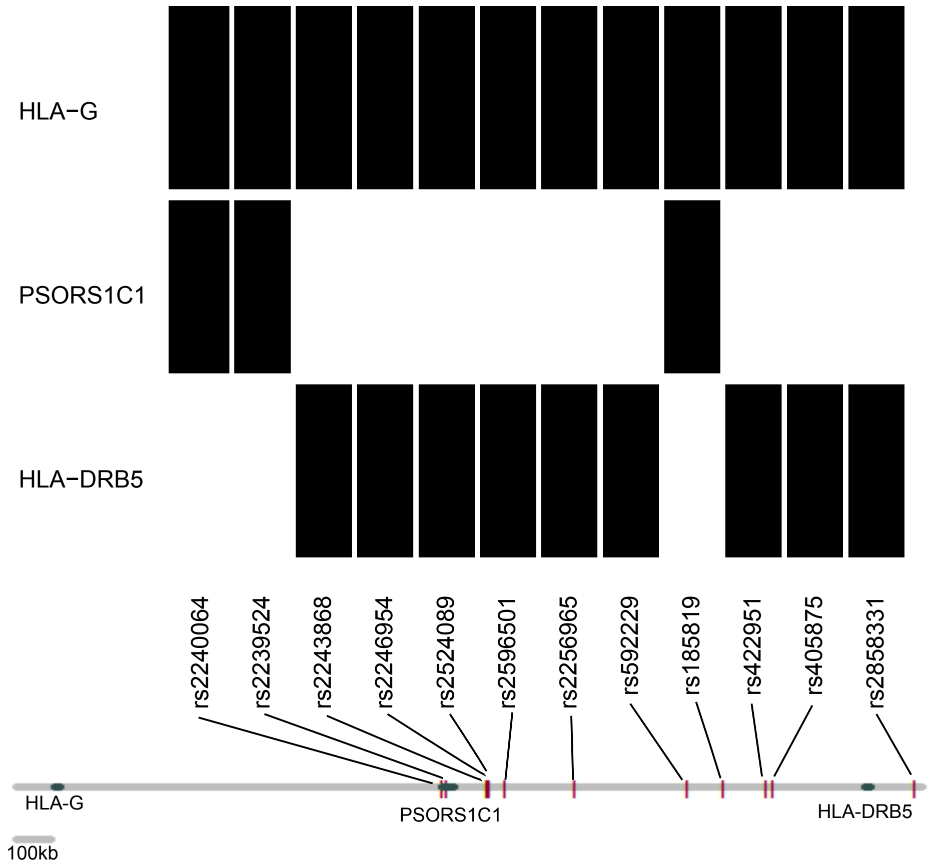

**Figure S15 - Correlated expression of genes under epistatic regulation in the pre-frontal cortex**

The expression of three trancripts, namely, *HLA-G*, *PSORS1C1* and *HLA-DRB5* on chromosome 6 are correlated. *PSORS1C1* expression is negatively correlated with *HLA-G* expression ($\rho$ = -0.21, p = 4.21 x $10^{-}7$) and *HLA-DRB5* expression is positively correlated with *HLA-G* expression ($\rho$ = 0.18, p = 1.83 x $10^{-}5$). The presence of multiples of the same gene is due to there being multiple expression probes for a single gene.

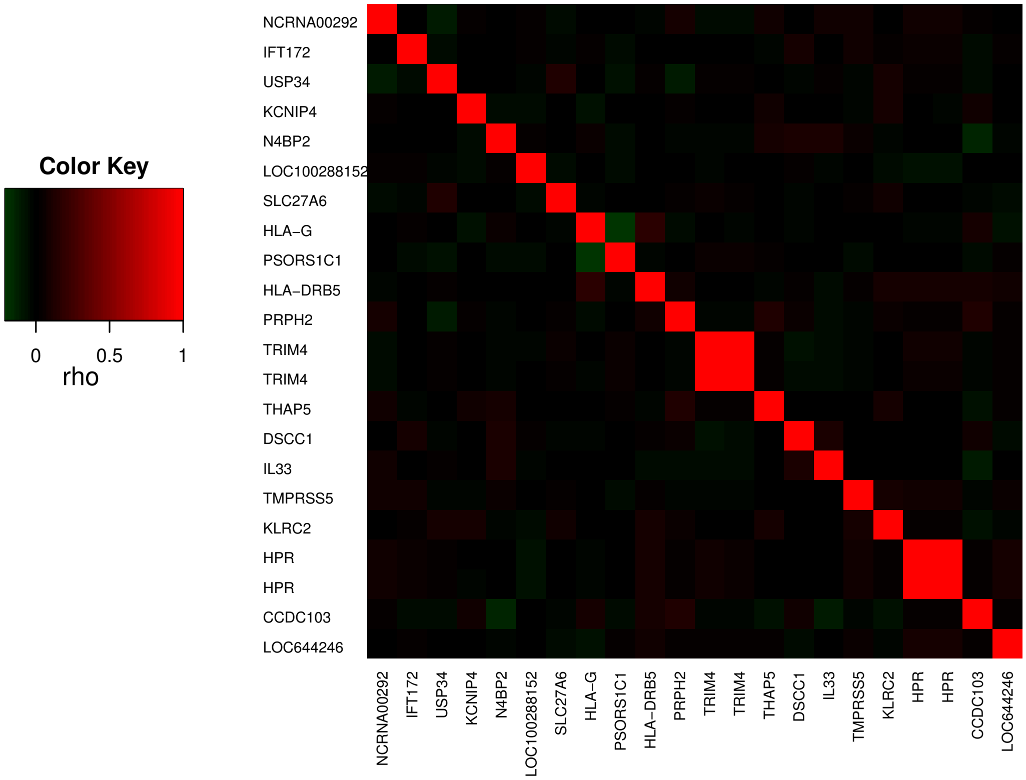

Supplement: Additional file 1: — Supplementary tables and figures. [file 12864_2015_1300_MOESM1_ESM.pdf]
